# A Portfolio Approach to Massively Parallel Bayesian Optimization

**DOI:** 10.1613/jair.1.16868

**Published:** 2025-01-14

**Authors:** Mickaël Binois, Nicholson Collier, Jonathan Ozik

**Affiliations:** Inria, Université Côte d’Azur, CNRS, LJAD, Sophia Antipolis, France; Consortium for Advanced Science and Engineering, University of Chicago Chicago, IL, USA; Argonne National Laboratory, Lemont, IL, USA; Consortium for Advanced Science and Engineering, University of Chicago Chicago, IL, USA

## Abstract

One way to reduce the time of conducting optimization studies is to evaluate designs in parallel rather than just one-at-a-time. For expensive-to-evaluate black-boxes, batch versions of Bayesian optimization have been proposed. They work by building a surrogate model of the black-box to simultaneously select multiple designs via an infill criterion. Still, despite the increased availability of computing resources that enable large-scale parallelism, the strategies that work for selecting a few tens of parallel designs for evaluations become limiting due to the complexity of selecting more designs. It is even more crucial when the black-box is noisy, necessitating more evaluations as well as repeating experiments. Here we propose a scalable strategy that can keep up with massive batching natively, focused on the exploration/exploitation trade-off and a portfolio allocation. We compare the approach with related methods on noisy functions, for mono and multi-objective optimization tasks. These experiments show orders of magnitude speed improvements over existing methods with similar or better performance.

## 1. Introduction

Current trends in improving the speed or accuracy of individual computations are on exploiting parallelization on highly concurrent computing systems. These computer models (a.k.a. simulators) are prevalent in many fields, ranging from physics to biology and engineering. Still, increasing the parallelization for individual simulators often comes with diminishing returns and model evaluation time remains limiting. A strategy is then to conduct several evaluations simultaneously, in batches, to optimize (here minimize) quantities of interest (see, e.g., Haftka et al., 2016, for a review).

For fast simulators, evolutionary algorithms (EAs) are amenable to parallelization by design, see, for instance, the review by Emmerich and Deutz (2018). But they require a prohibitive number of evaluations for more expensive-to-evaluate simulators. For these, Bayesian optimization (BO) (see, e.g., Shahriari et al., 2016, Frazier, 2018, Garnett, 2022) is preferred, with its ability to carefully select the next evaluations. Typically, BO relies on a Gaussian process (GP) model of the simulator, or any black-box, by using a probabilistic surrogate model to efficiently perform the so-called exploration/exploitation trade-off. Exploitation refers to areas where the prediction is low (for minimization), while exploration is for areas of large predictive variance. An infill criterion, or acquisition function, balances this trade-off to select evaluations, such as the expected improvement (EI) (Mockus et al., 1978) in the efficient global optimization algorithm (Jones et al., 1998). Alternatives include upper confidence bound (UCB) (Srinivas et al., 2010), knowledge gradient (Frazier, 2018), and entropy based criteria (Villemonteix et al., 2009b; Hennig & Schuler, 2012; Wang & Jegelka, 2017). Parallelization is then enabled by the definition of batch versions of the corresponding infill criteria, selecting several designs to evaluate at once.

Noisy simulators have their own set of challenges, as detailed in Baker et al. (2022), and raise questions about selecting the right amount of replication. While not necessary per se, repeating experiments is the best option to separate signal from noise, and is beneficial in terms of computational speed by limiting the number of unique designs, see, for example, Binois et al. (2019), Zhang et al. (2022). Rather than arbitrarily fixing the amount of replication a priori, allocating it adaptively is a way to improve accuracy for a given evaluation budget.

Here, we also consider multi-objective optimization (MOO) where the goal is to find the set of best compromise solutions, the Pareto front, since there is rarely a solution minimizing all objectives at once. We refer to Hunter et al. (2019) for a review of MOO options for black-boxes and Emmerich et al. (2020) for multi-objective (MO) BO infill criteria. MO versions of batch algorithms have also been proposed, taking different scalarization weights (Zhang et al., 2010), or relying on an additional notion of diversity (Lukovic et al., 2020).

Our motivating example is the calibration of a large-scale agent-based model (ABM) of COVID-19 run on a supercomputer (Ozik et al., 2021) with the added goal of reducing the *time to solution* for meaningful, i.e., timely, decision-making support. The targeted setup is as follows: a massively parallel system (for example, HPC cluster or supercomputer) with the ability to run hundreds of simulation evaluations in parallel over several iterations for the purpose of reducing the overall time to solution of the optimization to support rapid turnaround of analyses for high consequence decision making. Examples include public health (Ozik et al., 2021), meteorological (Goubier et al., 2020), and other emergency response (Mandel et al., 2019). This is sometimes called the high throughput regime (Hernández-Lobato et al., 2017) or scalable BO (Eriksson et al., 2019). Hence the time dedicated to select the batch points should be minimal (and not considered negligible), and this selection procedure amenable to parallelization (to use the available computing concurrency). While ABMs, or other noisy simulators, are applications where larger batch sizes are useful, this may also be the case for high-dimensional problems, even if larger evaluation budgets alone are not sufficient to avoid the curse dimensionality.

The method we propose is to directly identify candidates realizing different exploration/exploitation trade-offs. This amounts to approximating the GP predictive mean vs. variance Pareto front, which is orders of magnitude faster than optimizing most existing batch infill criteria. In doing so, we shift the paradigm of optimizing (or sampling) acquisition functions over candidate batches to quickly finding a set of desirable candidates to choose from. In the noisy setup, possibly with input-dependent noise variance, the predictive variance reduction makes an additional objective to further encourage replication. Then, to actually select batches, we follow the approach proposed by Guerreiro and Fonseca (2016) with the hypervolume Sharpe ratio indicator (HSRI) in the context of evolutionary algorithms. In the MO version, the extension is to take the predictive mean and variance of each objective, and still select batch-candidates based on the HSRI. The contributions of this work are: **1)** The use of a portfolio allocation strategy for batch-BO, defined on the exploration/exploitation trade-off. It extends directly to the multi-objective setup; **2)** An approach independent of the size of the batch, removing limitations of current batch criteria for large q; **3)** The potential for flexible batch sizes and asynchronous allocation via the portfolio approach; **4)** The ability to natively take into account replication and to cope with input-dependent noise variance.

In Section 2 we briefly present GPs, batch BO and MO BO as well as their shortcomings for massive batches. In Section 3, the proposed method is described. It is then tested and compared empirically with alternatives in Section 4. A conclusion is given in Section 5.

## 2. Background and Related Works

We consider the minimization problem of the black-box function f:

$$\text{find}\;x^{*}\in \underset{x\in X\subseteq R^{d}}{\text{argmin}}f(x)$$

where X is typically a hypercube. Among various options for surrogate modeling of f (see, e.g., Shahriari et al., 2016), GPs are prevalent.

### 2.1 Gaussian Process Regression

Consider a set of $n\in N^{*}$ designs-observations couples $\left(x_{i},y_{i}\right)$ with $y_{i}=f\left(x_{i}\right)+\varepsilon_{i},\varepsilon_{i}\sim 𝒩\left(0,\tau \left(x_{i}\right)\right)$, often obtained with a Latin hypercube sample as design of experiments (DoE). The idea of GP regression, or kriging, is to assume that f follows a multivariate normal distribution, characterized by an arbitrary mean function m(x) and a positive semi-definite covariance kernel function k:X×X→R. Unless prior information is available to specify a mean function, m is assumed to be zero for simplicity. As for k, parameterized families of covariance functions such as Gaussian or Matérn ones are preferred, whose hyperparameters (process variance $\sigma^{2}$, lengthscales) can be inferred in many ways, such as maximum likelihood estimation.

Conditioned on observations $y≔\left(y_{1},\ldots ,y_{n}\right)$, zero mean GP predictions at any set of q designs in $X,{𝒳}_{q}:{\left(x_{1}^{\prime },\ldots ,x_{q}^{\prime }\right)}^{⊤}$, are still Gaussian, $Y_{n}\left({𝒳}_{q}\right)|y\sim 𝒩\left(m_{n}\left({𝒳}_{q}\right),s_{n}^{2}\left({𝒳}_{q}\right)\right)$:

$$m_{n}\left({𝒳}_{q}\right)=k_{n}{\left({𝒳}_{q}\right)}^{⊤}K_{n}^{-1}y_{n},\;s_{n}^{2}\left({𝒳}_{q}\right)=k\left({𝒳}_{q},{𝒳}_{q}\right)-k_{n}{\left({𝒳}_{q}\right)}^{⊤}K_{n}^{-1}k_{n}\left({𝒳}_{q}\right)+\tau \left({𝒳}_{q}\right)$$

where $k_{n}\left({𝒳}_{q}\right)=({k{(x}_{i},x_{j}^{\prime }))}_{1\leq i\leq n,1\leq j\leq q}$ and $K_{n}=({k(x_{i},x_{j})+\delta_{i=j}\tau \left(x_{i}\right))}_{1\leq i,j\leq n}$. We refer to Rasmussen and Williams (2006), Forrester et al. (2008), Ginsbourger (2018), Gramacy (2020) and references therein for additional details on GPs and associated sequential design strategies.

Noise variance, τ(x), if present, is seldom known and must be estimated. With replication, stochastic kriging (Ankenman et al., 2010) relies on empirical noise variance estimates. Otherwise, estimation methods have been proposed, building on the Markov chain Monte Carlo method of Goldberg et al. (1998), as discussed, for example, by Binois et al. (2018). Not only is replication beneficial in terms of predictive variance estimation, it also has an impact on the computational speed of using GPs, where the costs scale with the number of unique designs rather than the total number of evaluations. This stems from the fact that p replicates $y_{1},\ldots ,y_{q}\sim 𝒩(f(x),\tau (x))$ at the same x are equivalent for GP prediction to a single observation $\overset{‾}{y}\sim 𝒩(f(x),\tau (x)/p)$.

### 2.2 Batch Bayesian Optimization

Starting from the initial DoE to build the starting GP model, (batch-) BO sequentially selects one (q) new design(s) to evaluate based on the optimization of an acquisition function that balances exploration and exploitation. The GP model is updated every time new evaluation results are available. The generic synchronous batch BO loop is illustrated in Algorithm 1. An asynchronous adaptation is detailed in Appendix A.

**Algorithm 1:** Pseudo-code for batch BO

**Require: $N_{\text{max}}$** (total budget), q (batch size), GP model trained on initial DoE ${\left(x_{i},y_{i}\right)}_{1\leq i\leq n}$	
1:	**while** $n\leq N_{\text{max}}$ **do**
2:	Choose $x_{n+1},\ldots ,x_{n+q}\in \text{arg}{\text{max}}_{{𝒳}_{q}\in X}\left({𝒳}_{q}\right)$
3:	Update the GP model by conditioning on ${\left\{x_{n+i},y_{n+i}\right\}}_{1\leq i\leq q}$.
4:	n←n+q
5:	**end while**

Among acquisition functions α, we focus on EI, with its analytical expression, compared to, say, entropy criteria. EI (Mockus et al., 1978) is defined as: $\alpha_{EI}(x)≔E\left[\text{max}\left(0,T-Y_{n}(x)\right)\right]$ where T is the best value observed so far in the deterministic case. In the noisy setup, taking T as the best mean estimation over sampled designs (Villemonteix et al., 2009a) or the entire space (Gramacy & Lee, 2011), are alternatives. Integrating out noise uncertainty is done by Letham et al. (2019), losing analytical tractability. This acquisition function can be extended to take into account the addition of q new points, with, for example, the batch (q in short) EI, $\alpha_{qEI}\left({𝒳}_{q}\right)≔E\left[\text{max}\left(0,T-Y_{n}\left(x_{1}^{\prime }\right),\ldots ,T-Y_{n}\left(x_{q}^{\prime }\right)\right]\right.$ that has an expression amenable for computation (Chevalier & Ginsbourger, 2013) and also for its gradient (Marmin et al., 2015).

A much faster approximation of the batch EI (qEI) is described by Binois (2015), relying on nested Gaussian approximations of the maximum of two Gaussian variables from Clark (1961). Otherwise, stochastic approximation (Wang et al., 2020) or sampling methods by Monte Carlo, with, for instance, the reparameterization trick (Wilson et al., 2018), are largely used, but may be less precise as the batch size increases. These methods may be combined for evaluating the parallel knowledge-gradient acquisition function, similar to EI but where the improvement is over the future optimum, see for instance Wu and Frazier (2016), Balandat et al. (2020). Using gradients may help as well (Wu et al., 2017), but they are seldom present in the noisy simulators.

Many batch versions of infill criteria have been proposed, such as Kandasamy et al. (2018), Hernández-Lobato et al. (2017) for Thompson sampling or Hernández-Lobato et al. (2017), Moss et al. (2021) for information-theoretic ones. For EI, rather than just looking at its local optima (Sóbester et al., 2004), some heuristics propose to select batch points iteratively, replacing unknown values at selected points by pseudo-values (Ginsbourger et al., 2010). This was coined as “hallucination” in the UCB version of Desautels et al. (2014). More generally, Rontsis et al. (2020) use an optimistic bound on EI for all possible distributions compatible with the same first two moments as a GP, which requires solving a semi-definite problem, limiting the scaling up to large batches. Gonzalez et al. (2016) reduce batch selection cost by not modeling the joint probability distribution of the batch nor using a hallucination scheme. Their idea is to select batch members sequentially by penalizing proximity to the previously selected ones.

Taking different infill criteria is an option to select different trade-offs, as by Tran et al. (2019), or with inter-distances as a secondary objective by Bischl et al. (2014). This idea of a portfolio of acquisition functions is also present in Hoffman et al. (2011), but limited to a few options and not intended as a mechanism to select batch candidates. This option is further enabled by Lyu et al. (2018) by randomly selecting from the Pareto front between acquisition functions. If these acquisition functions are the mean and predictive variance, then this leads to methods such as those proposed, for example, by Gupta et al. (2018), De Ath et al. (2021). Using local models is another way to select batches efficiently, up to several hundreds in Wang et al. (2018). The downside is a lack of coordination in the selection and the need of an *ad hoc* selection procedure. For entropy or stepwise uncertainty reduction criteria (see, e.g., Chevalier et al., 2014), batching would increase their already intensive computational burden. Another early work by Azimi et al. (2010) attempts to match the expected sequential performance, via approximations and sampling.

### 2.3 Multi-objective Bayesian Optimization

The multi-objective optimization problem (MOOP) is to find

$$x^{*}\in \underset{x\in X\subset R^{d}}{\text{argmin}}\left(f_{1}\left(x\right),\ldots ,f_{p}\left(x\right)\right),$$

p≥2.p>4 is often called the *many-objective* setup, with its own set of challenges for BO (see, e.g., Binois et al., 2020). The solutions of a MOOP are the best compromise solutions between objectives, in the Pareto dominance sense. A solution x is said to be dominated by another $x^{\prime }$, denoted $x^{\prime }⪯x$, if $\forall i,f_{i}\left(x^{\prime }\right)\leq f_{i}(x)$ and $f_{i}\left(x^{\prime }\right)<f_{i}(x)$ for at least one i. The Pareto set is the set of solutions that are not dominated by any other design in $X:\left\{x\in R^{d},∄x^{\prime }\in R^{d}\right.\;\text{such}\;\text{that}\;\left.x^{\prime }⪯x\right\}$; the Pareto front is its image in the objective space. In the noisy setup, we consider the noisy MOOP defined on expectations over objectives (Hunter et al., 2019).

**Figure 1:**
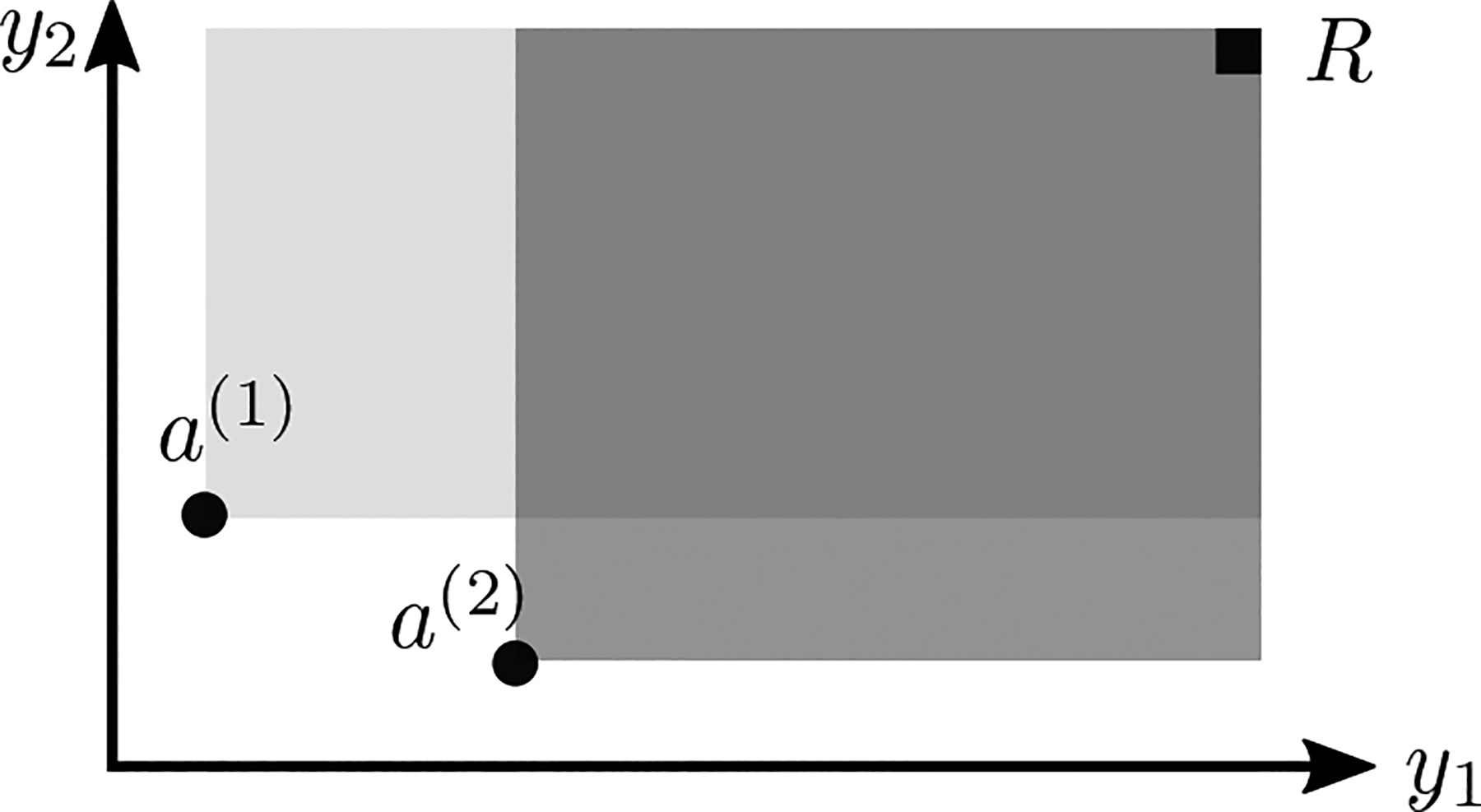
Hypervolume dominated by two assets $a^{\left(1\right)}$ (light gray) and $a^{\left(2\right)}$ (gray) with respect to the reference point R, corresponding to the expected return. The covariance return is given by the volume jointly dominated by both points (dark gray).

Measured in the objective space, the hypervolume refers to the volume dominated by a set of points relative to a reference point R, see Figure 1. It serves both as a performance metric in MOO, see, for example, Audet et al. (2021) and references therein, or to measure improvement in extending EI (Emmerich et al., 2006). The corresponding expected hypervolume improvement (EHI) can be computed in closed form for two or three objectives (Emmerich et al., 2011; Yang et al., 2017), or by sampling (see, e.g., Svenson, 2011). A batch version of EHI, qEHI, is proposed by Daulton et al. (2020, 2021). The operations research community has seen works dealing with low signal-to-noise ratios and heteroscedasticity, where replication is key. Generally, the idea is to first identify good points before defining the number of replicates, see, for example, Zhang et al. (2017), Gonzalez et al. (2020) or Rojas-Gonzalez and Van Nieuwenhuyse (2020) for a review on stochastic MO BO. Still, the batch aspect is missing in the identification of candidates.

### 2.4 Challenges with Massive Batches

There are several limitations in the works above. First, while it is generally assumed that the cost of one evaluation is sufficiently high to consider the time to select new points negligible, this may not be the case in the large batch setup. Parallel infill criteria are more expensive to evaluate, and even computational costs increasing linearly in the batch size (q) become impractical for hundreds or thousands of batch points. For instance, the exact qEI expression uses multivariate normal probabilities, whose computation do not scale well with the batch size. There are also many approximated criteria for batch EI, or similar criteria. However, in all current approaches, the evaluation costs increase with the batch size, at best linearly in q for existing criteria, which remains too costly for the regime we target.

This is already troublesome when optimization iterations must be conducted quickly, but is amplified by the difficulty of optimizing the acquisition function itself. While earlier works used branch and bounds (Jones et al., 1998) to guarantee optimality with q=1, multi-start gradient based optimization or EAs are predominantly used. In the batch setting, the size of the optimization problem becomes q×d, a real challenge, even with the availability of gradients. Wilson et al. (2018) showed that greedily optimizing batch members one-by-one is sensible, which still requires to solve qd-dimensional global optimization problems. Both become cumbersome for large batches and, presumably, far from the global optimum since the acquisition function landscape is multimodal, with flat regions, and symmetry properties. Plus, as we showcase, this results in parts of batch members being less pertinent.

Relying on discrete search spaces bypasses parts of the issue, even though finding the best batch becomes a combinatorial search. In between the greedy and joint options is the work by Daxberger and Low (2017), to optimize an approximated batch-UCB criterion as a distributed constraint problem. As a result, only sub-optimal solutions are reachable in practice for batch acquisition function optimization. Rather than optimizing, a perhaps even more computationally intensive option is to consider the density under EI. That is, to either find local optima and adapt batch size as in Nguyen et al. (2016), or sampling uniformly from the EI density with slice sampling and clustering as with Groves and Pyzer-Knapp (2018). Thompson sampling for mono-objective batch BO, see, for example, Hernández-Lobato et al. (2017), Kandasamy et al. (2018), also bypasses the issues of optimizing the acquisition function, but batch members are independently obtained, which can be wasteful for large batches or when uncertainty about the location of the optimizer is low. Lastly, adaptive batch sizes might be more efficient than a fixed number of parallel evaluations (see, e.g., Desautels et al., 2014). Similarly, asynchronous evaluation is another angle to exploit when the simulation evaluation times vary, see, for instance, Gramacy and Lee (2009), Janusevskis et al. (2012), Alvi et al. (2019).

Replication adds another layer of decision: whether or not adding a new design is worth the future extra computational cost, compared to the perhaps marginally worse option of replicating on the acquisition function value. With high noise, choosing the amount of replication becomes important, as individual evaluations contain almost no information. But even fixing the number of replicates per batch, selecting batch design locations plus replication degree makes a hard dynamic programming optimization problem.

## 3. Batch Selection as a Portfolio Problem

We propose an alternative to current BO methods to handle large batches by returning to the roots of BO, with the exploration/exploitation trade-off. The idea is to first identify batch candidates on this trade-off surface before selecting among them. Specifically, we focus on a portfolio selection criterion to select a batch balancing risk and return, while handling replication.

### 3.1 Exploration/Exploitation Trade-off

At the core of BO is the idea that regions of interest have either a low mean, or have a large predictive variance. This is the BO exploration/exploitation trade-off, see, for example, Garnett (2022). From a multi-objective point of view, acquisition functions resolve this trade-off by selecting a solution on the corresponding mean vs. standard deviation $\left(m_{n}/s_{n}\right)$ Pareto front 𝒫. With UCB (Srinivas et al., 2010), $\alpha_{UCB}(x)≔-m_{n}(x)+\sqrt{\beta }s_{n}(x)$, the tuning parameter β is a way to select one solution on the convex parts of this Pareto front, or a batch by taking several βs (see, e.g., Hutter et al., 2012). EI can be interpreted this way as well, as noticed by Jones et al. (1998), De Ath et al. (2021) in showing that

$$\frac{\partial EI}{\partial m_{n}}(x)=-\Phi \left(\frac{T-m_{n}(x)}{s_{n}(x)}\right)<0\;\text{and}\;\frac{\partial EI}{\partial s_{n}}(x)=\phi \left(\frac{T-m_{n}(x)}{s_{n}(x)}\right)>0,$$

where ϕ (resp. Φ) are the Gaussian pdf (resp. cdf). Hence EI also selects a specific solution on the corresponding Pareto front.

Navigating the Pareto front can be done by taking expectations of powers of the improvement, i.e., the generalized EI (GEI) (Schonlau et al., 1998; Wang et al., 2017), for which higher powers of EI reward larger variance and make it more global, as in Ponweiser et al. (2008). Note that the probability of improvement (PI), $\alpha_{PI}=P\left(Y_{n}(x)<T\right)$, which corresponds to the zeroth-order EI, is not on the trade-off Pareto front 𝒫, explaining why PI is often discarded as being too exploitative (higher variance is detrimental as soon as the predicted mean is below T).

Our main point is that rather than having to define a specific trade-off between exploration and exploitation *a priori*, before considering batching, it is better to find the set of optimal trade-offs 𝒫 and select batch points from it *a posteriori*. This batch candidate selection from 𝒫 can be interpreted as forming a portfolio, where the allocation correspond to the number of replicates of each 𝒫 design.

### 3.2 Portfolio Selection with HSRI

We propose to use a criterion to select solutions on the exploration-exploitation Pareto front, rather than doing so randomly as in Gupta et al. (2018). Yevseyeva et al. (2014) defined the hypervolume Sharpe ratio indicator (HSRI) to select individuals in MO EAs, with further study on their properties by Guerreiro and Fonseca (2016, 2020). Here, we borrow from this portfolio-selection approach, where individual performance is related to expected return, while diversity is related to the return covariance (interpreted as risk).

Let $A=\left\{a^{\left(1\right)},\ldots ,a^{\left(l\right)}\right\}$ be a non-empty set of assets, $a^{\left(i\right)}\in R^{s},s\geq 2$, let vector $r\in R^{l}$ denote their expected return and matrix $Q\in R^{l\times l}$ denote the return covariance between pairs of assets. Let $z\in [0,1{]}^{l}$ be the investment vector where $z_{i}$ denotes the investment in asset $a^{\left(i\right)}$. In portfolio assessment, an allocation can be evaluated in terms of expected return, $r^{⊤}z$ versus return variance $z^{⊤}Qz$, where higher returns come with more risk, hence forming a Pareto front. One classical way of selecting a solution is to consider the reward to volatility ratio, or Sharpe ratio. We refer the interested reader to Cornuejols and Tütüncü (2006) for more details and alternatives. The Sharpe-ratio maximization problem is defined as

(1) $$\underset{z\in [0,1{]}^{l}}{\text{max}}h(z)=\frac{r^{⊤}z-r_{f}}{\sqrt{z^{⊤}Qz}}\;\text{such}\;\text{that}\;\sum_{i=1}^{l}z_{i}=1,$$

with $r_{f}$ the return of a riskless asset and h the Sharpe ratio. This problem, restated as a convex quadratic programming problem (QP), see, for example, Cornuejols and Tütüncü (2006), Guerreiro and Fonseca (2016), can be solved efficiently only once per iteration:

(2) $$\zeta^{*}\in \text{arg}\;\underset{\zeta \in R^{l}}{\text{min}}\zeta^{⊤}Q\zeta \;\text{such}\;\text{that}\;\sum_{i=1}^{l}\left(r_{i}-r_{f}\right)\zeta_{i}=1,\zeta_{i}\geq 0,1\leq i\leq l.$$

The outcome is a set of weights, corresponding to the allocation to each asset, $z^{*}=\zeta^{*}/\sum_{i=1}^{l}\zeta_{i}^{*}$.

HSRI by Guerreiro and Fonseca (2016) is an instance of portfolio selection where the expected return and return covariance are based on the hypervolume improvement: $r_{i}=p_{ii}$ and $Q_{ij}=p_{ij}-p_{ii}p_{jj}$ where

(3) $$p_{ij}=\left(\prod_{1\leq t\leq p}\left(R_{l}-\text{max}\left(a_{t}^{\left(i\right)},a_{t}^{\left(j\right)}\right)\right)\right)/\left(\prod_{1\leq t\leq p}\left(R_{l}-f_{l}^{*}\right)\right);$$

see Figure 1 for an illustration. Note that this hypervolume computation scales linearly with the number of objectives. Importantly, as shown by Guerreiro and Fonseca (2016), if a set of assets is dominated by another set, its Sharpe ratio is lower. Furthermore, no allocation is made on dominated points: they are all on 𝒫. Finally they show that only the reference point R needs to be set in practice.

### 3.3 Proposition with qHSRI

From a BO viewpoint, the goal is to obtain the q candidates leading to the highest return in terms of HSRI:

$$\alpha_{\text{qHSRI}}\left({𝒳}_{q}\right)=h\left(z\left({𝒳}_{q}\right)\right)=\left(r{\left({𝒳}_{q}\right)}^{⊤}1_{q}-r_{f}\right){\left(\sqrt{1_{q}^{⊤}Q\left({𝒳}_{q}\right)1_{q}}\right)}^{-1}$$

with $1_{q}$ a vector of q ones. Here, instead of using actual objective values as in MO EAs, an asset $a^{\left(i\right)}$, corresponding to candidate design $x^{\left(i\right)}$, is characterized by its GP predictive mean(s) and standard deviation(s), i.e., with $p=1,a_{1}^{\left(i\right)}=m_{n}\left(x^{\left(i\right)}\right)$ and $a_{2}^{\left(i\right)}=-s_{n}\left(x^{\left(i\right)}\right)$. Since the hypervolume metric is not invariant to a rescaling of the objectives, standard deviations rather than variances are used to define the Pareto front 𝒫 to have comparable quantities across the objectives. Also here $r_{f}=0$ since risk-less assets (noiseless observations whose predictive uncertainty is already zero) bring no improvement.

Directly optimizing $\alpha_{\text{HSRI}}$ would have the issues raised in Section 2.4: too many optimization variables and a significant computational cost, from repeated QP problem solvings. But since the optimal solutions will belong to 𝒫 thanks to the properties of HSRI, the search can be decomposed into three steps: approximating 𝒫, solving the QP problem (2) over this approximation of 𝒫, and then selecting q evaluations. In the noiseless case, they are the q largest weights. When replication is possible, the allocation becomes proportional to the weights:

$$\text{find}\;\gamma \;\text{such}\;\text{that}\;\sum_{i=1}^{l}\left\lfloor \gamma \times z_{i}^{*}\right\rceil =q,$$

by bisection (randomly resolving ties). The adaptation to the asynchronous setup is detailed in Appendix A.

#### Extension to the Multi-objective Setup

To extend to MO, we propose to search trade-offs on the objective means, $m_{n}^{\left(1\right)}(x),\ldots ,m_{n}^{\left(p\right)}(x)$, and averaged predictive standard deviations Pareto front,

$${\overset{‾}{\sigma }}_{n}(x)=p^{-1}\sum_{i=1}^{p}s_{n}^{\left(i\right)}(x)/\sigma_{n}^{\left(i\right)}$$

with ${\left(\sigma_{n}^{\left(i\right)}\right)}^{2}$ the $i^{\text{th}}$-objective GP variance hyperparameter, a p+1 dimensional space. Taking all p standard deviations is possible, but the corresponding objectives are correlated since they increase with the distance to observed design points. In the case where the GP hyperparameters are the same and evaluations of objectives coupled, the objectives would be perfectly correlated. Even with different hyperparameters, preliminary tests showed that using the averaged predictive standard deviation do not degrade the performance compared to the additional difficulty of handling more objectives.

#### Replication

When noise is present, we include an additional objective of variance reduction. That is, for two designs with the same predictive mean and variance, the one for which adding an observation will decrease the predictive variance the most is preferred. This decrease is given by GP update equations (see, e.g., Chevalier et al., 2014) and does not depend on the value at the future q designs:

(4) $$s_{n+q}^{2}\left({𝒳}_{q}\right)=s_{n}^{2}\left({𝒳}_{q}\right)-s_{n}^{2}\left({𝒳}_{q},x_{1:(n+q)}\right){\left(s_{n}^{2}\left(x_{1:(n+q)}\right)\right)}^{-1}s_{n}^{2}\left(x_{1:(n+q)},{𝒳}_{q}\right),$$

with $x_{1:(n+q)}$ defined as the current DoE augmented by future q designs. It does depend on the noise variance and the degree of replication, see, for example, Binois et al. (2019), which may be used to define a minimal degree of replication at candidate designs to ensure a sufficient decrease. Similarly, it is possible to limit the replication degree when the decrease of predictive variance of further replication is too low.

The pseudo-code of the approach is given in Algorithm 2. The first step is to identify s designs on the mean vs. standard deviation Pareto set. In the deterministic case s must be at least equal to q, while with noise and replication it can be lower. Population based evolutionary algorithms can be used here, with a sufficiently large population. In general, the number of non-dominated solutions found would be large enough, especially when including an archive. For massive batches with the deterministic setting, which is not the envisioned main use of qHSRI, an appropriate stopping criterion could be used to ensure that the cardinality of 𝒫 is larger than q. Once these s candidates are identified, dominated points can be filtered out as HSRI only selects non-dominated solutions. Points with low probability of improvement (or with low probability of being non-dominated) can be removed as well. This prevents the method to be over-exploratory.

In the noisy setup, the predictive variance reduction serves as an additional objective for the computation of HSRI. It is integrated afterwards as it is not directly related to the identification of the exploration-exploitation tradeoff surface. Computing qHSRI then involves computing r and Q before solving the corresponding QP problem (1). Designs from $X_{s}$ are selected based on $z^{*}$: either by taking the q designs having the largest weights $z_{i}$, or computing γ to obtain an allocation, which can include replicates.

**Algorithm 2:** Pseudo-code for batch BO with qHSRI

**Require:** $N_{\text{max}}$ (total budget), q (batch size), p GP model(s) fitted on initial DoE ${\left({\text{x}}_{i},y_{i}\right)}_{1\leq i\leq n}$	
1:	**while** $n\leq N_{\text{max}}$ **do**
2:	Find $X_{s}\in \text{argmin}\left(m_{n}^{\left(1\right)}(x),\ldots ,m_{n}^{\left(p\right)}(x),{\bar{\sigma }}_{n}(x)\right)$
3:	Filter dominated solutions in $X_{s}$
4:	**if p=1 then**
5:	Filter points with low PI in $X_{s}$
6:	**else**
7:	Filter points with low PND in $X_{s}$
8:	**end if**
9:	If τ(x)>0: add predictive variance reduction to objectives, Equation (4)
10:	Compute return r and covariance matrix Q using Equation (3)
11:	Compute optimal Sharpe ratio $z^{*}=\zeta^{*}/\sum_{i=1}^{l}\zeta_{i}^{*}$ by solving QP problem (2)
12:	Allocate q points based on the weights, see Section 3.3
13:	Update the GP model(s) with ${\left\{x_{n+i},y_{n+i}\right\}}_{1\leq i\leq q}$.
14:	n←n+q
15:	**end while**

In terms of complexity, the main task is to find the assets, i.e., candidate designs on 𝒫. Evaluating $m_{n}$ and $s_{n}$ cost $𝒪\left(n^{2}\right)$ after a $𝒪\left(n^{3}\right)$ matrix inversion operation that only needs to be done once. An order of magnitude can be gained with approximations, see for instance Wang et al. (2019). Then r and Q are computed on the assets, before maximizing the Sharpe ratio, whose optimal weights provide the best q designs. Filtering solutions reduces the size of the QP problem to be solved, either with PI or the probability of non-domination (PND) in the MO case. Crucially, the complexity does not depend on q with replication.

**Figure 2:**
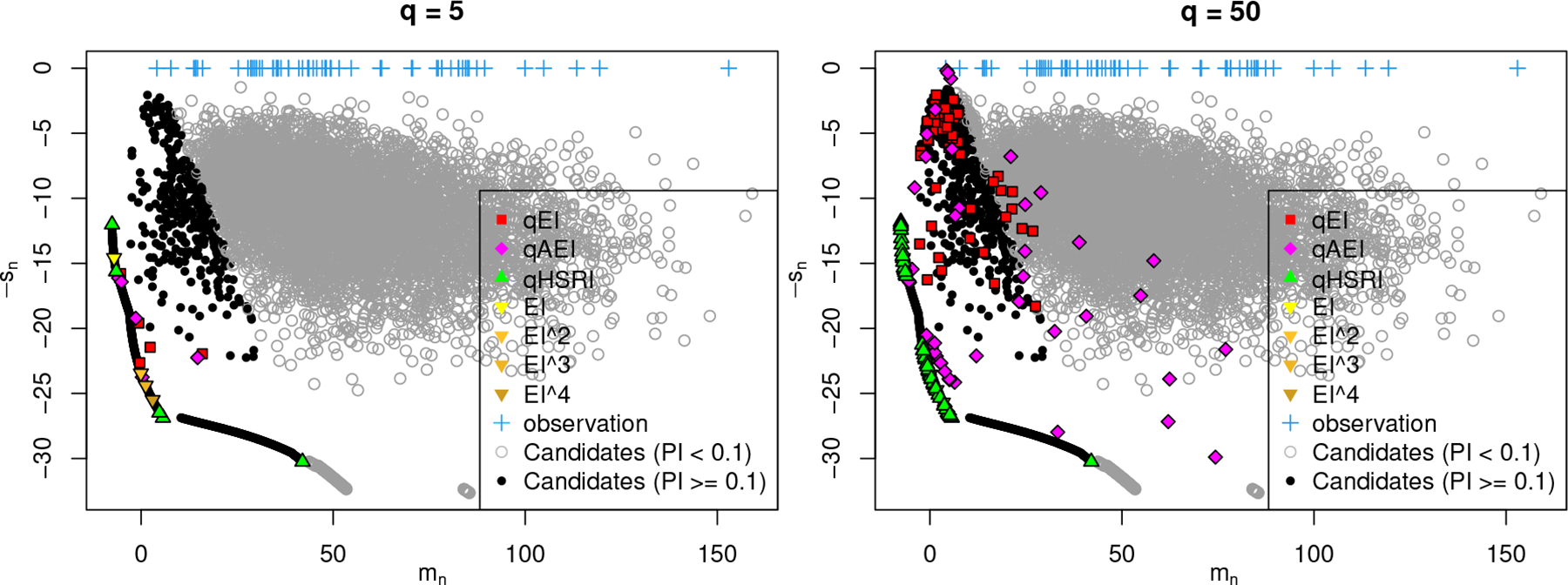
Comparison of qHSRI with qEI and qAEI acquisition functions on the noiseless repeated Branin function (d=6,n=60). The first four GEI optimal solutions are depicted as well.

We illustrate the proposed method (qHSRI) on an example in Figure 2, comparing the outcome of optimizing qEI, its fast approximation qAEI (Binois, 2015) and qHSRI in the mean vs. standard deviation ($m_{n}$ vs. $s_{n}$) space. Negation of the standard deviation is taken for minimization. Additional candidates are shown, either randomly sampled in X or on the estimated exploration/exploitation Pareto set. Interestingly, there is a gap between the two sets, indicating that uniformly sampling may be insufficient if used as a discrete optimization scheme. While all qHSRI selected designs are on 𝒫, this is not the case for the qEI version, particularly so when q is larger, where none of the selected designs are – possibly due to the much higher difficulty of solving the corresponding optimization problem. Designs corresponding to powers of EI also appear on 𝒫, showing a richer exploration/exploitation trade-off than with EI only. But they are not evenly spread since fixed a priori without adaptation to the shape of 𝒫. Observations (crosses) are not on 𝒫, as it is possible to find designs with better mean (unless exactly at the optimum) or larger predictive variance (increasing with the distance to observation). We also observe that points with large predictive variance may not be of interest if they have a negligible PI (for instance, < 0.1). In the experiments we use 1/3 as default value, while preliminary experiments indicate a low sensitivity to this parameter. A takeaway is that when dealing with large batches, all aspects must be considered simultaneously: optimization difficulty, adaptability to the shape of 𝒫, and computational speed.

It could be argued that the qHSRI approach ignores distances in the input space and could form clusters. While this is the case, depending on the exploration-exploitation Pareto set, since the batch points cover 𝒫, it automatically adapts to this latter’s range of optimal values, depending on the iteration and problem. This is harder to adapt *a priori* in the input space and it avoids having to define minimal distances manually, as in Gonzalez et al. (2016). Still, for numerical stability and to favor replication, a minimal distance can be set as well.

#### Convergence

For the mono-objective version, the convergence analysis is related to the one employed in the GP-UCB strategy by Gupta et al. (2018) since points on the 𝒫 can be associated with β values. I.e., if a suitable $\beta_{0}$ is included in the selected candidates, where other β values are higher, then the algorithm follows the standard sublinear convergence for such methods. For the multi-objective version, we can similarly rely on the results of Paria et al. (2020) on UCB for random scalarizations of the objectives. Again, the selected candidates on the 𝒫 can be associated with a scalarization counterpart and convergence guarantees follow if the corresponding β is large enough.

## 4. Experiments

Except Wang et al. (2018) that uses disconnected local GP models and Gupta et al. (2018) that also uses the exploration-exploitation PF, existing batch BO methods mostly give results with low q, with typically q≤20. On the implementations we could test, these criteria take more than a few seconds per evaluation with q≈100, while, in our approach, predicting for a given design takes less than a millisecond. Some approaches, like by Eriksson et al. (2019) or Lukovic et al. (2020), could scale better but are not adapted to handle low signal-to-noise regimes. Consequently, comparisons with qHSRI are not always feasible for massive q, especially for noisy problems. We thus limit q to 50 in comparisons, while scaling results for larger q values with qHSRI, up to 500, are provided in Appendix B.

The R package hetGP (Binois & Gramacy, 2021) is used for noisy GP modeling. Anisotropic Matérn covariance kernels are used throughout the examples, whose hyperparameters are estimated by maximum likelihood. As we use the same GP model, the comparison shows the effect of the acquisition function choice: qEI or qEHI vs. qHSRI. qEI is either the exact version (Chevalier & Ginsbourger, 2013) in DiceOptim (Picheny et al., 2021), or the approximated one from Binois (2015), qAEI. qEI is not available for q>20 nor in the noisy setup. In the mono-objective case, we also include Thompson sampling, qTS, implemented by generating GP realisations on discrete sets of size 200d. qEHI is from GPareto (Binois & Picheny, 2019), estimated by Monte Carlo. PF denotes the method from Gupta et al. (2018), where the batch members are randomly selected on the exploration-exploitation Pareto front. Random search (RS) is added as a baseline, as well as R package mlrMBO (Bischl et al., 2017) (MBO) for which we use the default values. These correspond to the use of different β values with UCB in the mono-objective case, complemented by an hypervolume indicator in the multi-objective case (available in the deterministic setup only). All start with the same space filling designs of size 5d, replicated five times each to help the heteroscedastic GP modeling with low signal-to-noise ratios.

Optimization of the acquisition functions is performed by combining random search, local optimization and EAs. That is, for qEI, $n_{u}=100d$ designs are uniformly sampled in the design space before computing their univariate EI. Then $n_{b}=100d$ candidate batches are created by sampling these designs with weights given by EI. Then the corresponding best batch for qEI is optimized locally with L-BFGS-B. A global search with pso (Bendtsen, 2012) (population of size 200) is conducted too, to directly optimize qEI, and the overall best qEI batch is selected. The same principle is applied for qEHI. As for qHSRI and PF, in addition to the same uniform sampling strategy with $n_{u}$ designs, NSGA-II (Deb et al., 2002) from mco (Mersmann, 2020) is used to find 𝒫, the exploration/exploitation compromise surface (with a population of size 500). The reference point R for HSRI computations is obtained from the range over each component, extended by 20%, as is the default in GPareto (Binois & Picheny, 2019). The R code (R Core Team, 2023) of the approach is available as supplementary material.

For one objective, the optimality gap, i.e., the difference to a reference solution, is monitored. With noise, the optimality gap is computed both on noiseless values (when known) and on the estimated minimum by each method over iterations, which is the only element accessible in real applications. In fact, the optimality gap only assesses whether a design has been sampled close to an optimal one, not if it has been correctly predicted. As we will see, it may be difficult to correctly identify the best solution among evaluations. The hypervolume metric is used in the MO case, from a reference Pareto front computed using NSGA-II and all designs found by the different methods. Similar to the mono-objective case, the hypervolume difference is also computed on the estimated Pareto set by each method, over iterations, to have a more reliable and realistic performance monitoring.

In a first step, for validating qHSRI, we compared it with alternatives for relatively low q values. These preliminary experiments on noiseless functions are provided in Appendix B. The outcome is that qHSRI gives results on par with qEI and qEHI looking at the performance over iterations, at a much lower computational cost. These results also motivate the use of qAEI as a proxy for qEI when this latter is not available. MBO outperforms the other methods with q=25 for the mono-objective problems iteration-wise, but is underperforming for multi-objective ones. It shows intermediate running times, better than qAEI or qTS but worse than PF or qHSRI, and it worsen with larger batches. We notice that qTS performed poorly on these experiments, possibly because using discretized realisations is insufficient for the relatively large input dimension (d=12). There the use of random or Fourier features may help (Mutny & Krause, 2018). As for PF, it requires more samples than qHSRI, even if it goes as fast. This indicates that the portfolio allocation strategy is beneficial compared to randomly sampling on the exploration-exploitation Pareto front.

### 4.1 Mono-objective Results

We first consider the standard Branin and Hartmann6 test functions, see, for example, Roustant et al. (2012). For the noisy Branin (resp. Hartmann6), we take the first objective of the P1 test function (Parr, 2012) (resp. repeated Hartmann3, Roustant et al., 2012) as input standard deviation $\tau (x{)}^{\frac{1}{2}}$, hence with heteroscedastic noise (denoted by *het* below).

**Figure 3:**
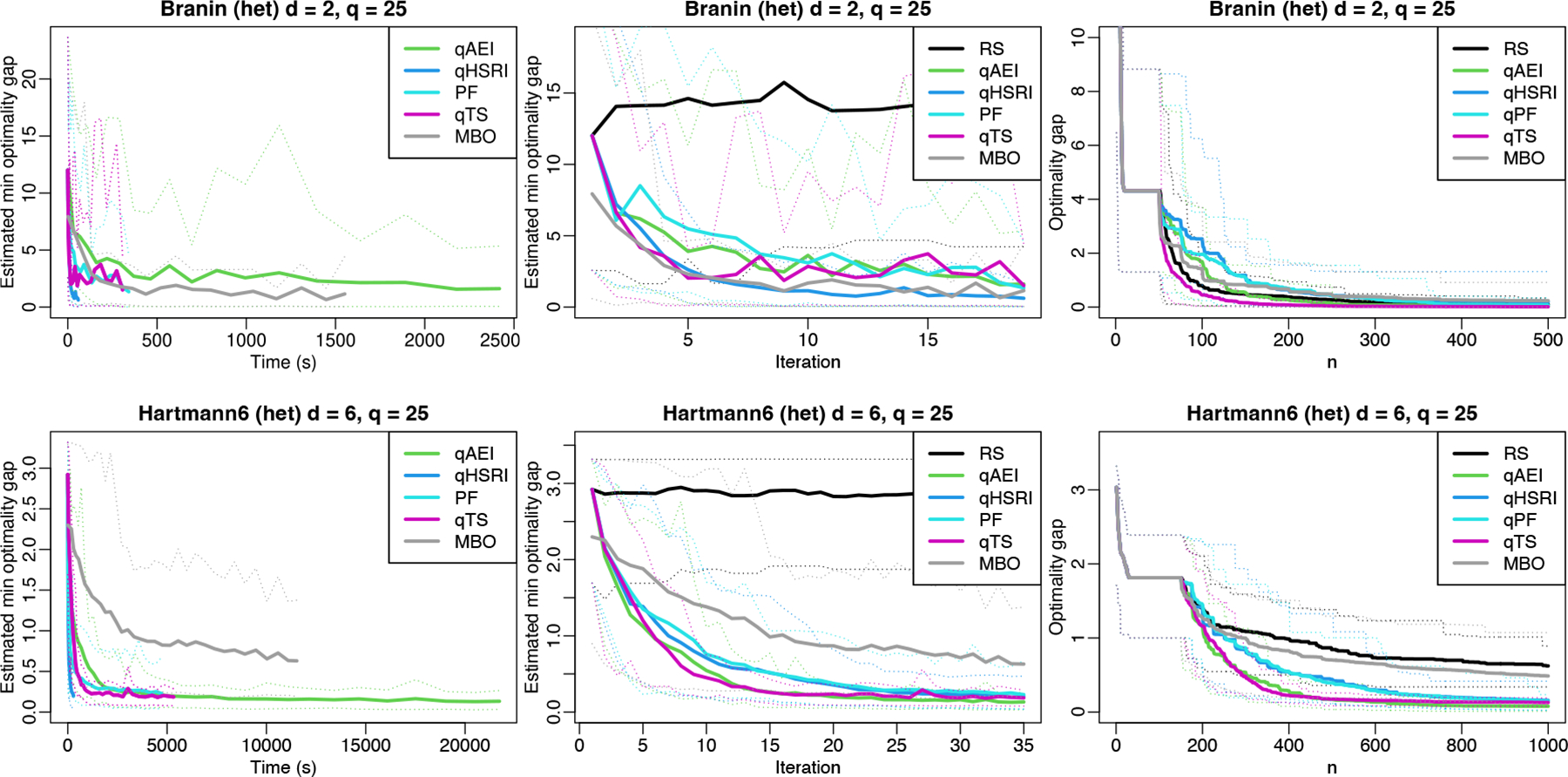
Mono-objective results over iterations and over time. Optimality gap and estimated optimality gap for noisy tests over 40 macro-runs are given. Thin dotted lines are 5% and 95% quantiles.

Figure 3 highlights that qHSRI is orders of magnitude faster than qTS, qAEI or MBO for decreasing the estimated optimality gap (see also Table 1 for all timing results). It also improves over random selection on 𝒫 as with PF. In these noisy problems, looking at the true optimality gap for observed designs shows good performance of RS, since, especially in small dimension like for Branin (d=2), there is a high probability of sampling close to one of its three global minima. Also, replicating incurs less exploration, penalizing qHSRI on this metric.

This is nevertheless misleading: in practice one cannot compute the true optimality gap since the true optimum is usually unknown. A surrogate’s predicted value for a design close to the optimum may not be identified as such. The actual metric of interest is the optimality gap of the estimated best solution at each iteration. It is improved with qHSRI, especially for Branin, while performances of the various acquisition functions are similar iteration-wise in the Hartmann6 case, with qTS being slightly better initially. For RS, the estimated best solution is the best value observed, which is driven by the noise realizations. The contrast with the optimality gap is particularly sharp for the Branin test case, since RS performs well under this metric. MBO struggles with noisy observations, especially for the Hartmann6 problem, and does not seem suitable for problems with low signal-to-noise.

Concerning speed, part of the speed-ups are due to the ability to replicate. Indeed, as apparent in supplementary Figure 9, for qHSRI the number of unique designs remains low, less than 20% of the total number of observations without degrading the sample efficiency. It directly reduces the BO time per iteration, see supplementary Figure 10. Notice that taking larger batches can be faster since the batch selection is independent of q with qHSRI. Also there are fewer iterations for the same simulation budget, hence less time is spent in fitting GPs and finding 𝒫.

Next we tackle the more realistic 12d Lunar lander problem (Eriksson et al., 2019). We take $N_{max}=2000$ with q=100, where a single evaluation is taken as the average over 100 runs to cope with the low signal-to-noise ratio (rather than fixing 50 seeds to make it deterministic as by Eriksson et al., 2019). The solution found (predicted by the GP) is of −205.32 while the reference handcrafted solution gives −101.13, see Figure 5. The Lunar lander problem with qHSRI took 5 hours; it did not complete with qAEI even in 24 hours due to q=100.

### 4.2 Multi-objective Results

**Figure 4:**
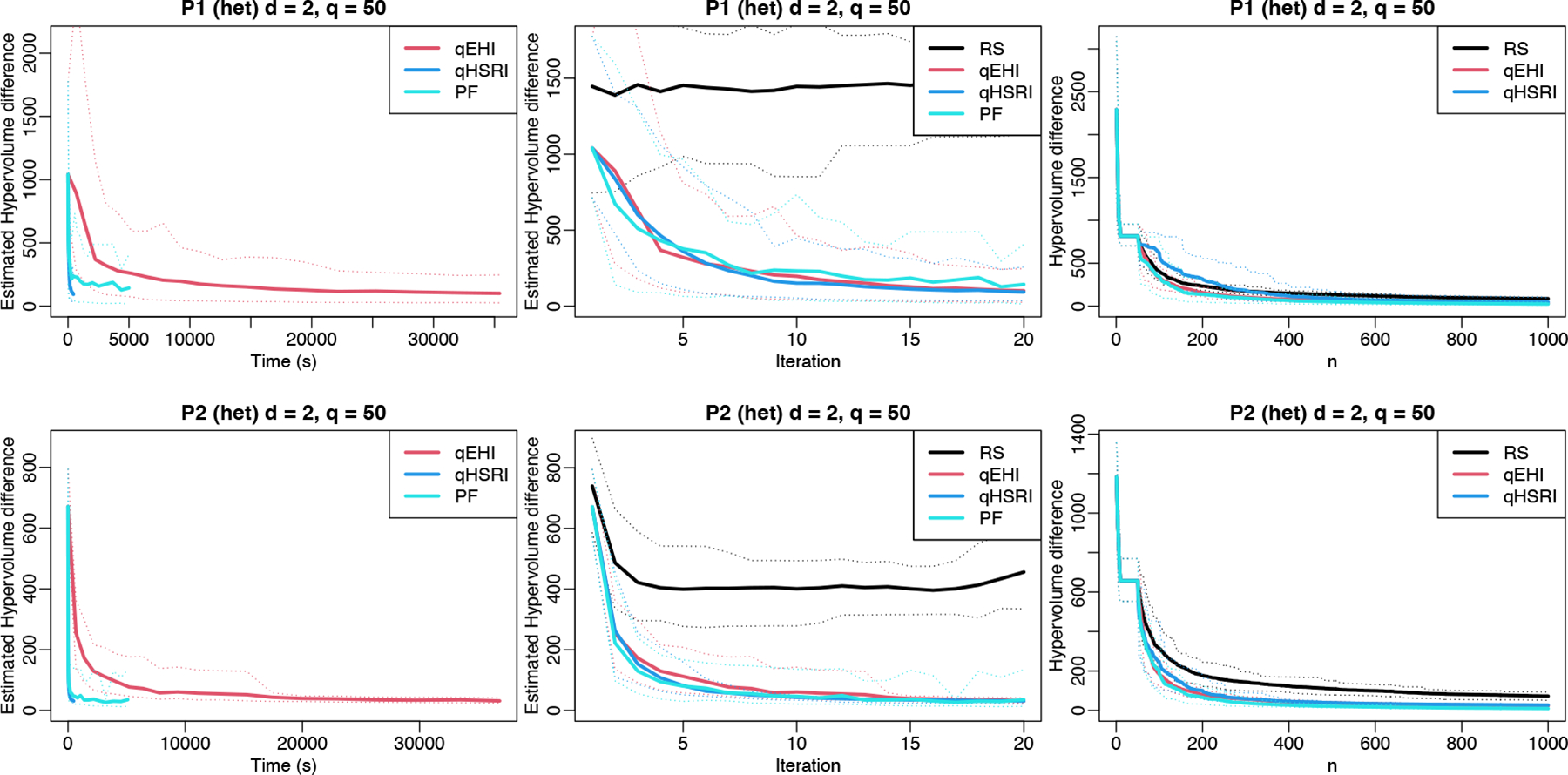
Multi-objective results over time, iterations, and number of samples. Hypervolume difference over a reference Pareto front and its counterpart for the estimated Pareto set for noisy tests over 40 macro-runs are given. Thin dotted lines are 5% and 95% quantiles.

We consider the P1 (Parr, 2012) and P2 (Poloni et al., 2000) problems. For the noisy setup, one problem serves as the other one’s noise standard deviation function (taking absolute values for positiveness). The results are shown in Figure 4, where the leftmost panels show the beneficial performance of the qHSRI approach in terms of time to solution. While RS performs relatively well looking solely at the hypervolume difference for the evaluated designs (rightmost panels), this does not relate to the quality of the Pareto front estimation. Indeed, the estimated Pareto with RS, that is, using only the non-dominated noisy observations, is far from the actual Pareto front, due to noise realizations. There the Pareto fronts estimated by GP models do show a convergence to the reference Pareto front, indicating that their estimation improves over iterations (middle panels). Finally, like in the mono-objective results, we demonstrate in Appendix B that for the noiseless case the sample efficiency of qHSRI is at least on par to that of qEHI, and even slightly better in some cases.

**Figure 5:**
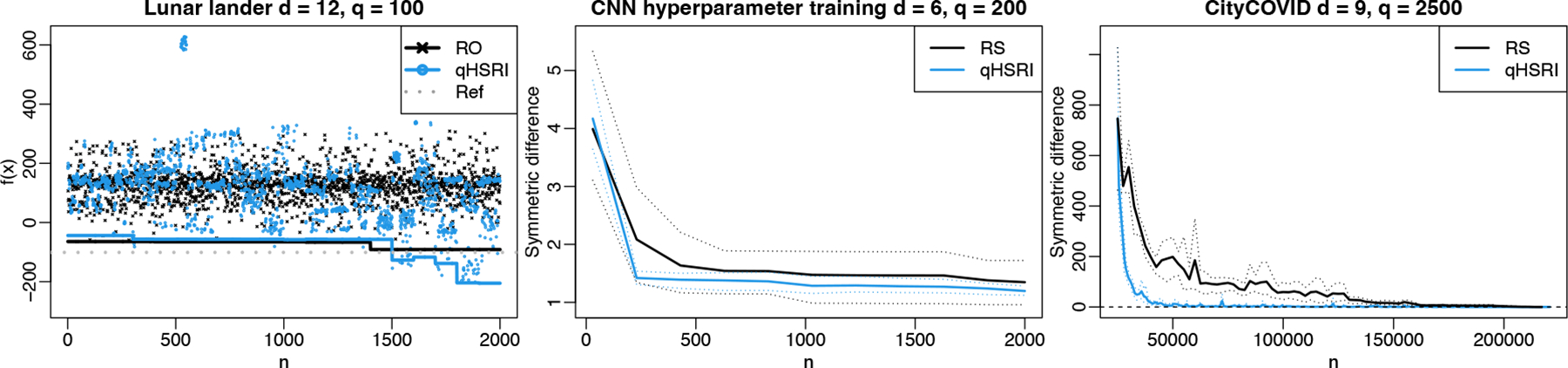
Left: optimality gap for the Lunar lander problem (one single run) with the evaluated values and estimated minimum found. Center: results of the tuning of the hyperparameters of a convolutional neural network over 5 macro-runs. Right: results on CityCOVID data set over 5 macro-runs. Thin dotted lines are 5% and 95% quantiles.

Next, we consider the training of a convolutional neural network (CNN) used for the classification of digits based on the MNIST data (LeCun et al., 1998), with 70,000 handwritten digits (including 10,000 for testing). There are six variables on this problem, detailed in Appendix C, modifying the neural network architecture and training. Neurons are randomly cut off during training to increase robustness, referred to as dropout, which introduces randomness to the process. The goal is to find compromise solutions between accuracy on the testing set and the CNN training time, with $N_{max}=2000$ and q=200. The outcome is presented in Figure 5, showing the symmetric difference between the estimated Pareto front over iterations and a reference Pareto front obtained by fitting a GP to all the observations collected. qSHRI consistently learns an accurate Pareto front faster than random search, even if the latter found good solutions in one macro-run.

### 4.3 CityCOVID Data Set

We showcase a motivating application example for massive batching: calibrating the CityCOVID ABM of the population of Chicago in the context of the COVID-19 pandemic, presented by Ozik et al. (2021) and built on the ChiSIM framework (Macal et al., 2018). It models the 2.7 million residents of Chicago as they move between 1.2 million places based on their hourly activity schedules. The synthetic population of agents extends an existing general-purpose synthetic population (Cajka et al., 2010) and statistically matches Chicago’s demographic composition. Agents colocate in geolocated places, which include households, schools, workplaces, etc. The agent hourly activity schedules are derived from the American Time Use Survey and the Panel Study of Income Dynamics and assigned based on agent demographic characteristics. CityCOVID includes COVID-19 disease progression within each agent, including differing symptom severities, hospitalizations, and age-dependent probabilities of transitions between disease stages.

The problem is formulated as a nine variable bi-objective optimization problem: the aggregated difference for two target quantities. It corresponds to the calibration of the CityCOVID parameters θ listed in Table 4, each normalized to [0, 1]. Model outputs are compared against two empirical data sources obtained through the City of Chicago data portal (City of Chicago, 2022): H the daily census of hospital beds occupied by COVID-19 patients and D the COVID-19 attributed death data in and out of hospitals, both for residents of Chicago. We used an exponentially weighted error function $L\left(\theta ,T_{i},{\tilde{T}}_{i},d\right),i\in \{H,D\}$, with daily discount rate d tuned to 98% and 95% for H and D, with the corresponding observed (resp. simulated) time-series denoted by T and $\tilde{T}$ giving the objectives.

To inform public health policy, many simulations are necessary in a short period of time, which can only be achieved by running many concurrently. One simulation takes ≈ 10min, with a low signal-to-noise ratio. A data set of 217, 078 simulations (over 8, 368 unique designs, with a degree of replication between 1 and 1000) has been collected by various strategies: IMABC (Rutter et al., 2019), qEHI with fixed degree of replication, and replicating non-dominated solutions. This data set is available in the supplementary material.

Contrary to the previous test cases that were defined over continuous domains, for testing qHSRI we use this existing data set. The initial design is a random subset of the data: 25, 000 simulation results over 2500 unique designs with a degree of replication of 10, out of 50, 585 simulations over 5075 unique designs, with a degree of replication between 3 and 10. These correspond to results given by IMABC (akin to a non uniform sampling). qHSRI is used to select candidates among remaining designs up to q=2500 if enough replicates are available, hence with a flexible batch size. To speed up prediction and to benefit from a parallel architecture, local GPs are built from 20 nearest neighbors rather than relying on a single global GP. We show in Figure 5 the progress in terms of symmetric difference to the final estimate of the Pareto front, thus penalizing both under and over confident predictions. qHSRI quickly converges to the reference Pareto front, compared to RS.

## 5. Conclusions and Perspectives

Massive batching for BO comes with the additional challenge that batch selection must happen at a fast pace. Here we demonstrate qHSRI as a flexible and light-weight option, independent of the batch size. It also handles replication natively, resulting in additional speed-up for noisy simulators without fixing a degree of replication. Hence, this proposed approach makes a sensible, simple, yet efficient, baseline for massive batching.

Randomness in simulators is often controlled by a seed. When the seed carries some information, it may be relevant to take it into account in the optimization (see, e.g., Pearce et al., 2022). For example, this could guide the selection of seeds when replicating. Possible extensions could take into account global effects (on entropy, integrated variance, etc.) of candidate designs to be less myopic. A more dynamic Sharpe ratio allocation could be beneficial, to improve replication. Additionally, while the integration of a few constraints is straightforward, handling more could rely on the use of copulas as in Eriksson and Poloczek (2021) to alleviate the increase of the dimension of the exploration/exploitation trade-off surface. Finally, based on results for UCB with various β values, future work could improve the convergence analysis to account for the observation that the requirements are often too conservative in practice (see, e.g., Paria et al., 2020).

## Supplementary Material

PBO Code

## Figures and Tables

**Figure 6:**
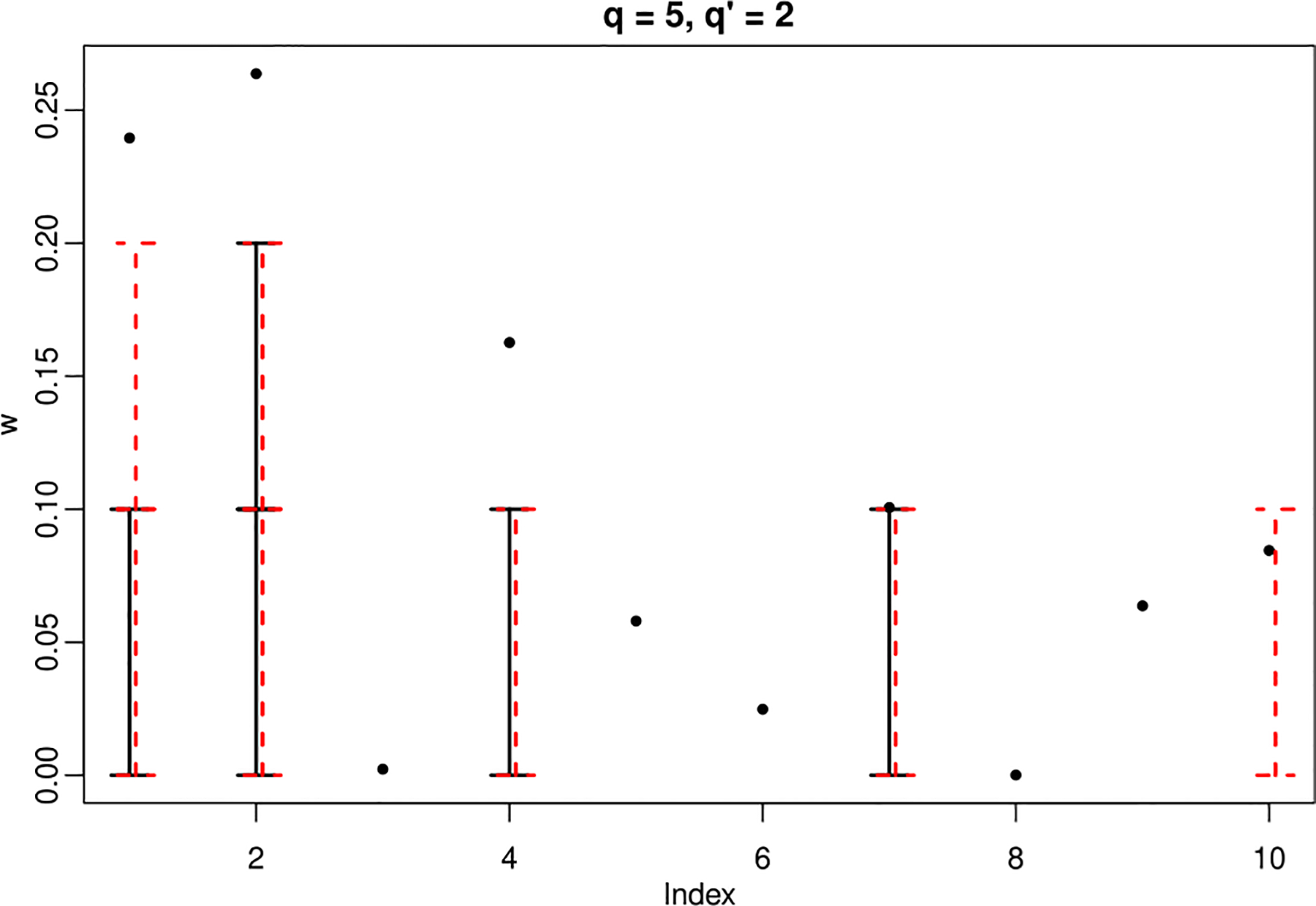
Batch allocation on indices according to weights w (black points) for 10 candidates. q=5 designs are initially allocated, as represented by the black segments. If $q^{\prime }=2$ additional designs can be evaluated, then the allocation is recomputed with the same weights and leads to the red dashed segments.

**Figure 7:**
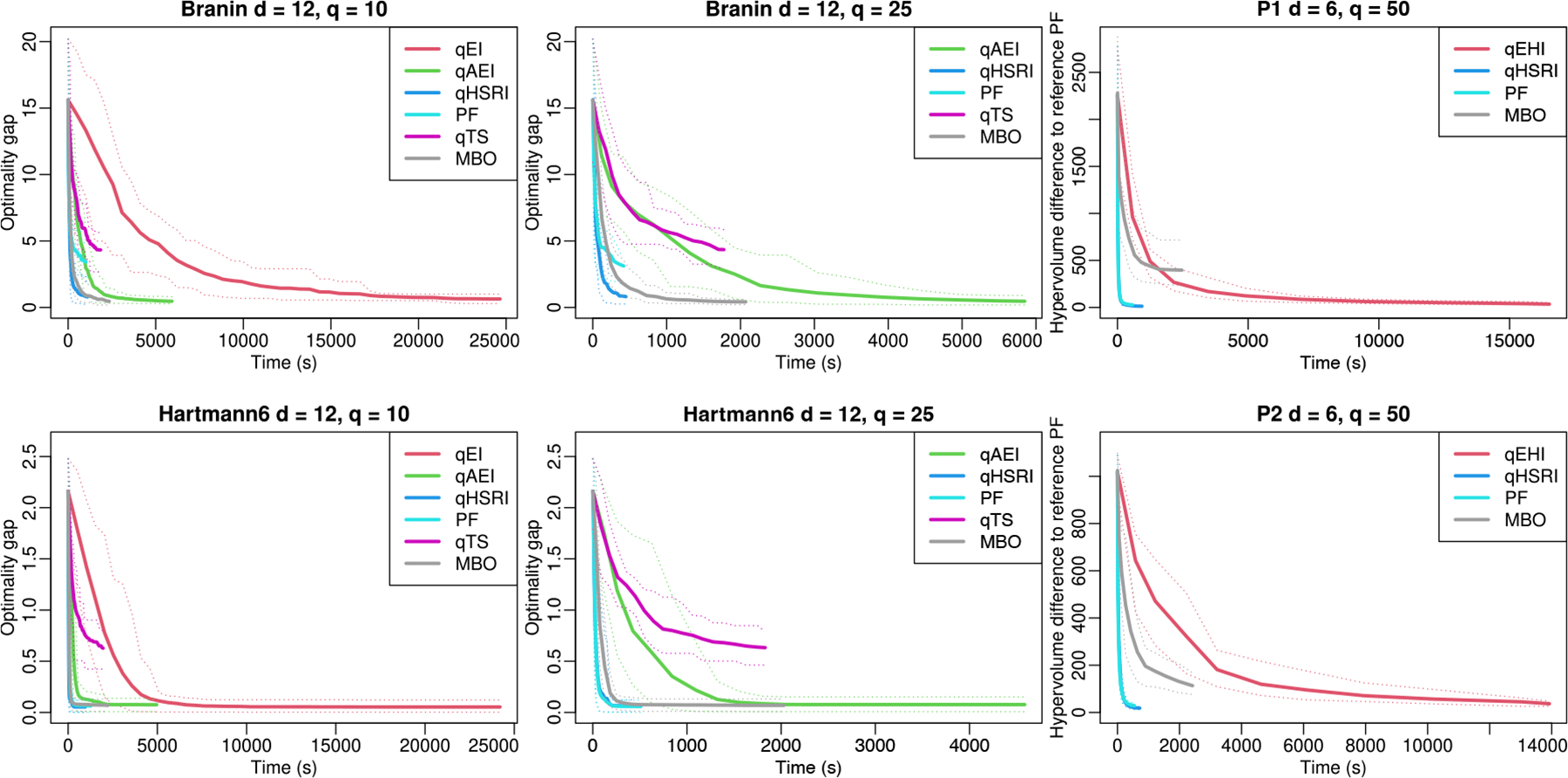
Noiseless results over time. Optimality gap or hypervolume difference over 20 macro-runs is given. Thin dotted lines are 5% and 95% quantiles.

**Figure 8:**
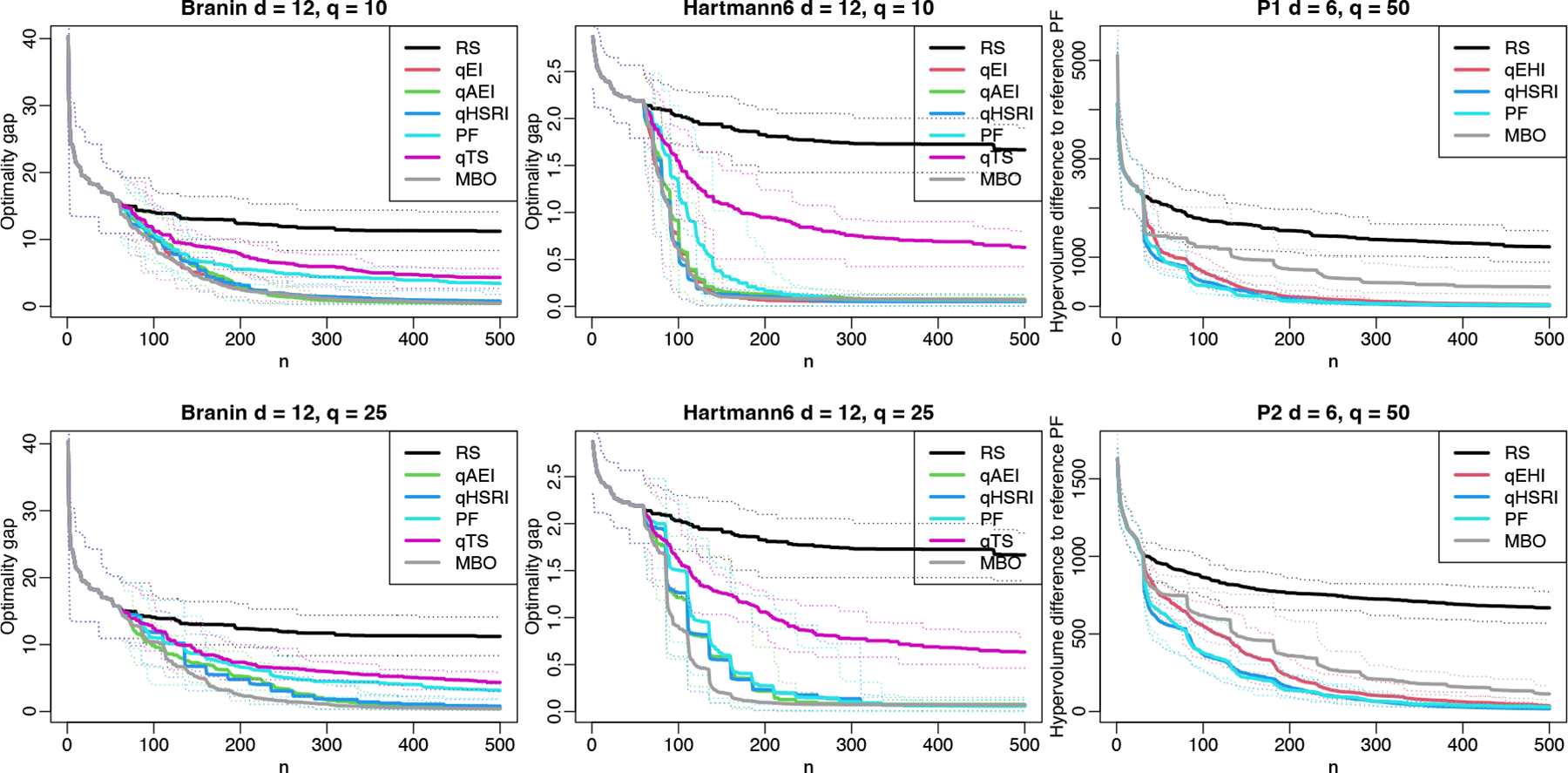
Noiseless results over iterations. Optimality gap or hypervolume difference over 20 macro-runs is given. Thin dotted lines are 5% and 95% quantiles.

**Figure 9:**
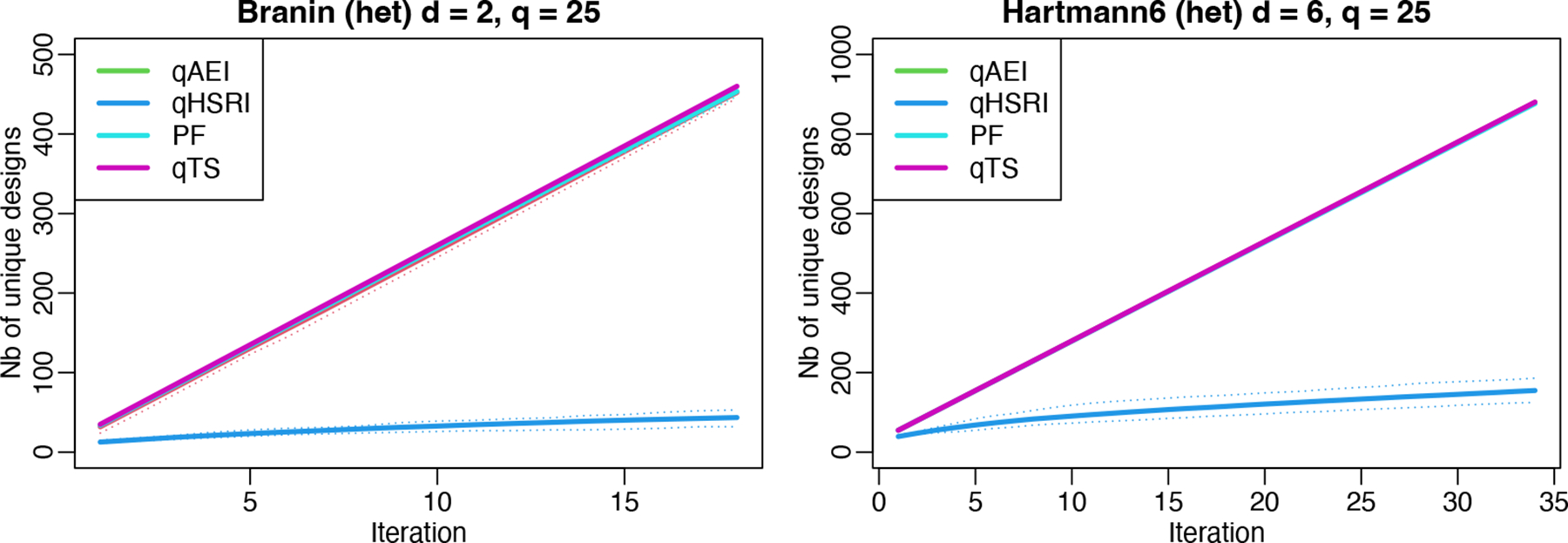
Number of unique designs over iterations for the mono-objective test problems. Thin dotted lines are 5% and 95% quantiles.

**Figure 10:**
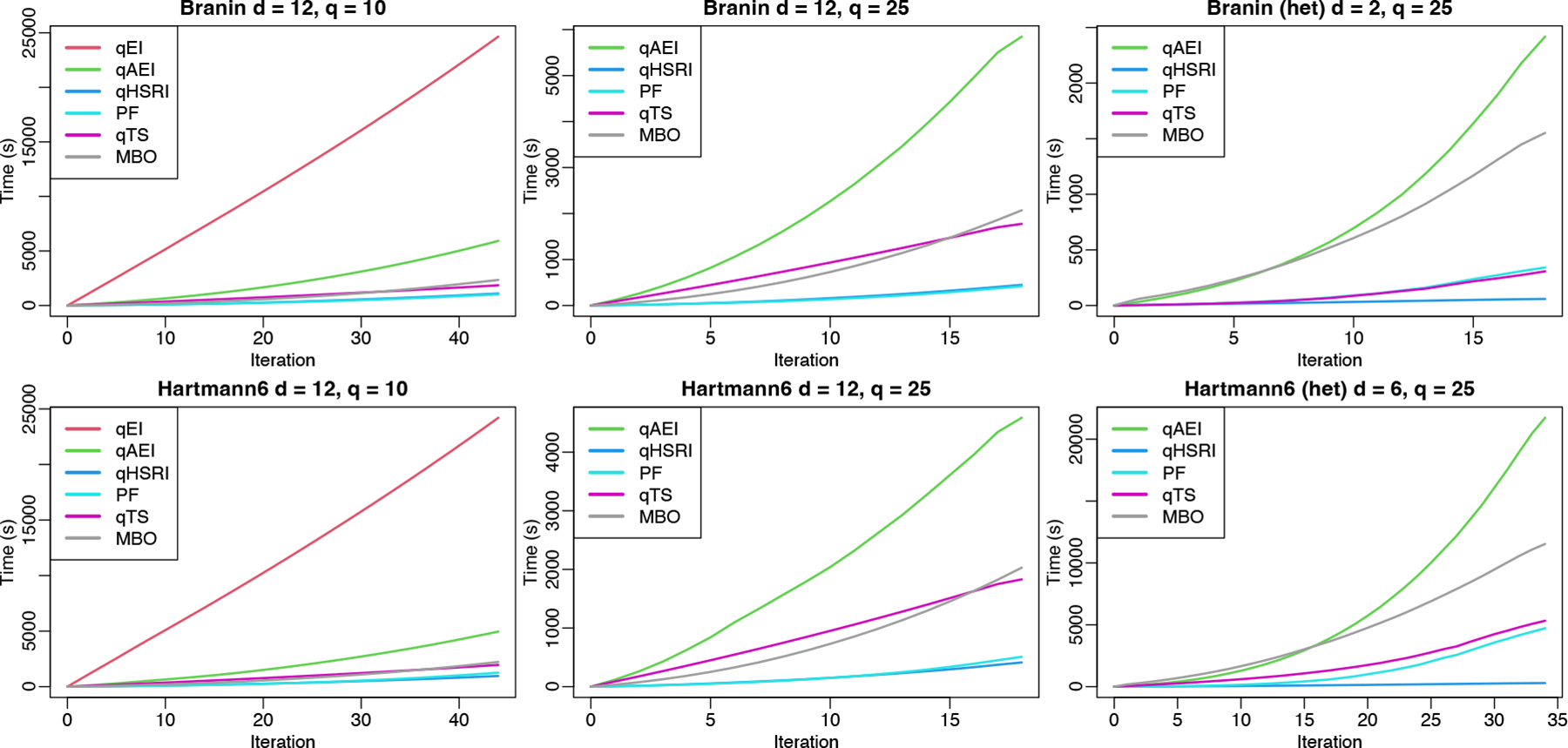
Time taken per iteration for the mono-objective test problems.

**Figure 11:**
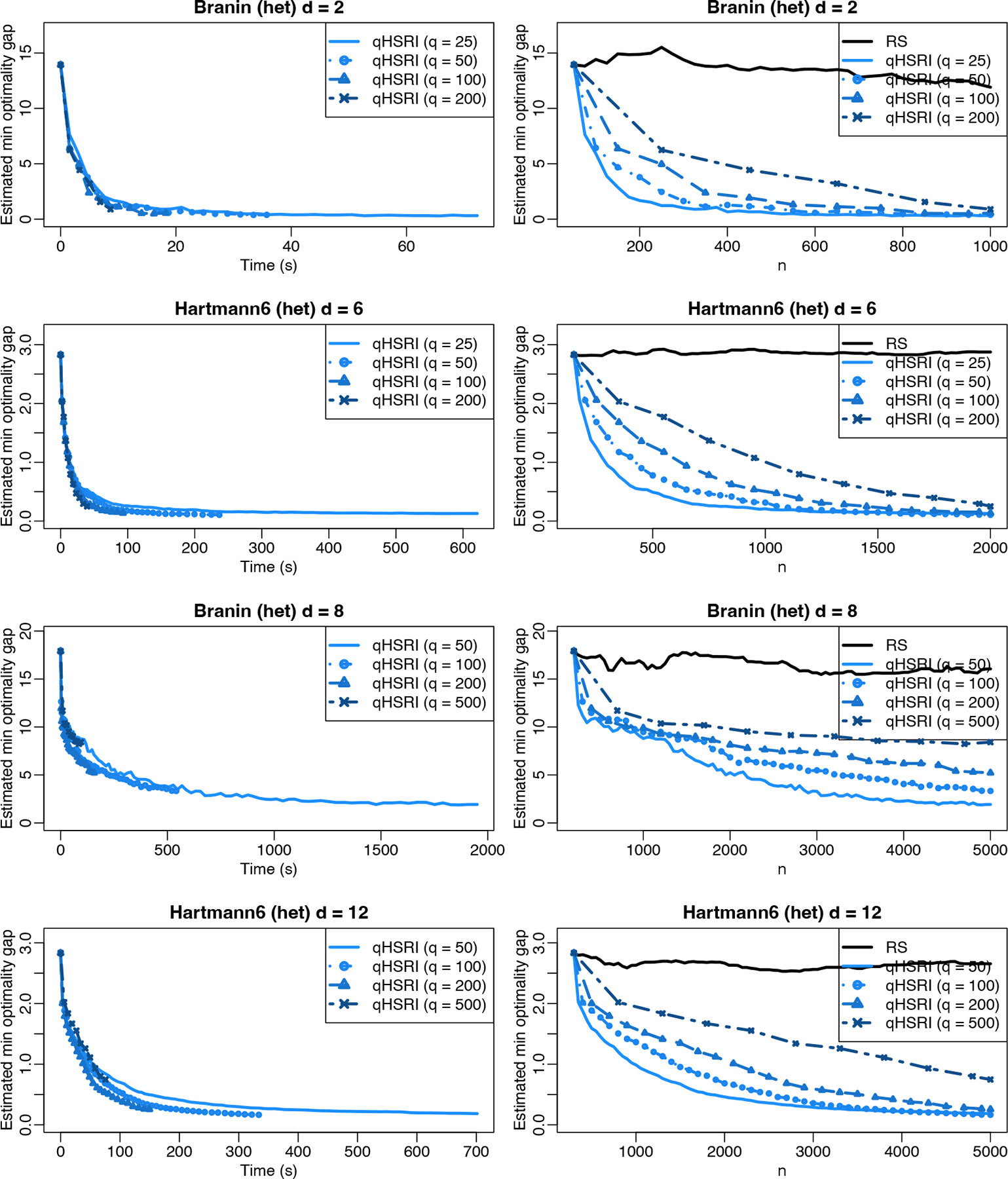
Mono-objective results over iterations and over time. Optimality gap and estimated optimality gap for noisy tests over 60 macro-runs are given.

**Figure 12:**
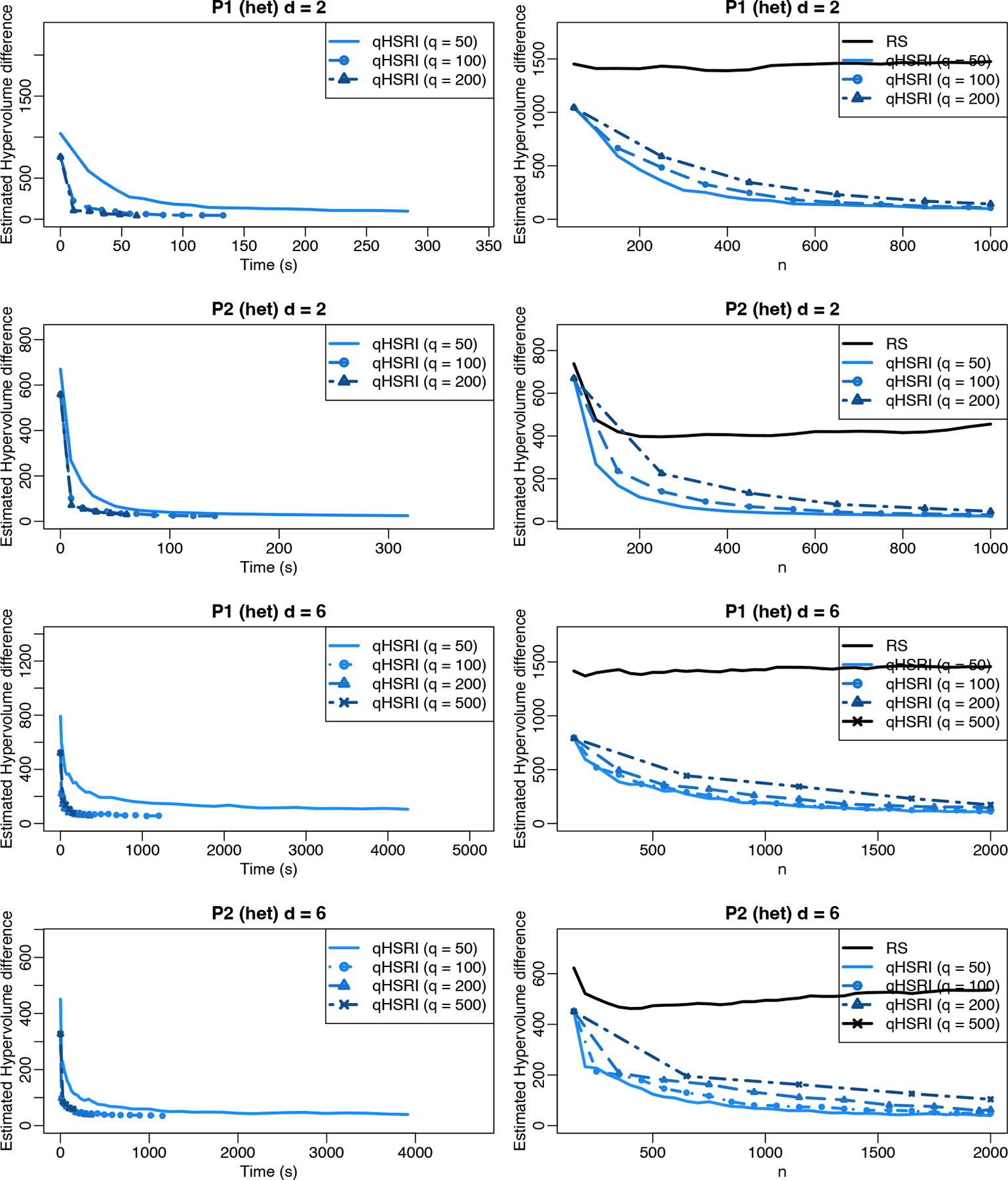
Multi-objective results over time and iterations. Hypervolume difference over a reference Pareto front and its counterpart for the estimated Pareto set for noisy tests over 60 macro-runs are given.

## Appendix A. Adaptation to Asynchronous Batch Bayesian Optimization

The standard batch- (or multi-points) BO loop, where parallel evaluations are conducted in parallel and waiting for all of them to finish, is presented in Algorithm 1. In the general asynchronous setup, new evaluations can be performed without waiting for all the batch results. The change is for the acquisition function, that needs to take into account running candidates in the selection, typically relying on pseudo-values for these, see, for example, Ginsbourger et al. (2010), Desautels et al. (2014). Next we discuss briefly the adaptations required for qHSRI in the asynchronous setup.

The main feature of the portfolio approach is to give a weight vector corresponding to Pareto optimal points on the mean/standard deviation Pareto front 𝒫. In the synchronous setting, q points are selected based on the weights. That is, the q best ones in the noiseless setting while replicates can occur in the noisy setup. If $q^{\prime }$ additional points can be evaluated, without new data becoming available (or while waiting for new candidates to evaluate), the change in the asynchronous setup amounts to considering that $q+q^{\prime }$ batch points (assets) can be selected according to the weights. The q first ones will remain unchanged while $q^{\prime }$ additional ones can be run. An example is provided in Figure 6.

## Appendix B. Additional Experimental Results

We complement the results of Section 4 on noisy problems with results on noiseless ones. The R package DiceKriging (Roustant et al., 2012) is used for deterministic GP modeling. In this deterministic setup, d is increased to accommodate larger batches via repeated versions of these problem as used, for example, by Oh et al. (2018).

The timing results (to complete all iterations) for all synthetic benchmarks are presented in Tables 1 and 2, with best results in bold. qHSRI or PF are always much faster than the alternatives, even the approximated qEI criterion (qAEI) or qTS. The R code (R Core Team, 2023) of the approach and the CityCOVID data are available as supplementary material. Results have been obtained in parallel on dual-Xeon Skylake SP Silver 4114 @ 2.20GHz (20 cores) and 192 GB RAM (or similar nodes). Lunar lander tests have been run on a laptop.

**Table 1:** Averaged timing results (in s), with standard deviation in parenthesis. B is for Branin, H for Hartmann6, (h) for heteroscedastically noisy problems and ‘–’ indicates when non applicable.

f−d−q	qEI	qAEI	qHSRI	qPF	qTS	MBO
B-12-10	24725 (133)	5901 (54)	1103 (104)	**1024** (160)	1835 (5)	2345 (35)
B-12-25	–	5856 (61)	451 (46)	**423** (66)	1760 (3)	2070 (16)
H-12-10	24258 (115)	4957 (131)	**969** (38)	1266 (144)	1938 (22)	2224 (25)
H-12–25	–	4585 (129)	**419** (24)	510 (76)	1816 (9)	2029 (9)
B(h)-2-25	–	2470 (93)	**57** (3)	342 (36)	305 (46)	1552 (56)
H(h)-6-25	–	22012 (717)	**288** (33)	4714 (702)	5327 (503)	11532 (408)

**Table 2:** Averaged timing results (in s), with standard deviation in parenthesis. (h) is for heteroscedastically noisy problems.

f−d−q	qEHI	qHSRI	qPF	MBO
P1-6-50	16308 (2118)	678 (25)	**580** (26)	2463 (20)
P1(h)-2-50	35350 (1363)	**567** (59)	5110 (407)	–
P2-6-50	14022 (1818)	635 (24)	**553** (20)	2419 (24)
P2(h)-2-50	37386 (1123)	**608** (44)	5148 (414)	–

The detailed results of the performance over time are given in Figure 7, where qHSRI show better results than the alternatives, closely matched by PF except on Branin where it does worse. qTS performs badly as the discretization is insufficient given the problem dimension. MBO shows an intermediate performance, especially for larger batch sizes.

The results over iterations of Figure 8 shows that the performance of qHSRI matches the one of qEI and improves over qEHI. qTS underperforms but it is still better than RS. PF shows similar results to qHSRI on the multi-objective problems and Hartmann6 with q=25, but is worse in the other test cases. For the smallest batch size, q=10, qAEI matches the performance of qEI, while being faster. MBO is best on the mono-objective q=25 case, but the multi-objective version is outperformed.

To highlight that replication occurs, Figure 9 represents the number of unique designs over time for the mono-objective noisy problems. Notice that replication is present in the initial design and that replication can happen for all methods when sampling vertices of the hypercube. This directly translates in the time spent per iteration to select batch candidates, see Figure 10. qEI is largely the most time consuming approach since the criterion is computationally expensive, followed by qAEI. Then MBO and qTS have more or less the same cost in the deterministic setup, while MBO is more expensive when the problem is noisy, possibly due to reinterpolation. PF and qHSRI have similar costs in the deterministic setup while qHSRI is much faster, especially for noisy problems, as it relies on replication.

We complement the synthetic experiments in Section 4 with results for larger values of q using qHSRI. The mono-objective results are in Figure 11 and the multi-objective ones in 12. Increasing q shows degrading results sample-wise, as there are fewer updates of the GP model. This effect is less pronounced on the multi-objective examples, since candidates can be spread along the Pareto set. But increasing q decreases the time to solution. Selecting an appropriate value of q for a given application thus involves taking into account the available parallelism, simulation time, signal-to-noise ratio, number of variables, and number of objectives.

## Appendix C. Details on the CNN Training Problem Parameters

The six variables of the CNN are given in Table 3. The architecture is composed of two 2D convolutional + max pooling layers, before two dense layers with dropout. The reference point used for hypervolume computations is [0, 150]. The CNN training is performed on GeForce GTX 1080 Ti GPUs.

**Table 3:** CNN network problem parameters description.

x	Lower bound	Upper bound	Description
$x_{1}$	2	100	Number of filters of first convolutional layer
$x_{2}$	2	100	Number of filters of second convolutional layer
$x_{3}$	0	0.5	First dropout rate
$x_{4}$	0	0.5	Second dropout rate
$x_{5}$	10	200	Number of units of dense hidden layer
$x_{6}$	2	50	Number of epochs

## Appendix D. Details on CityCOVID Calibration Parameters

The nine variables of the simulator are given in Table 4.

**Table 4:** CityCOVID calibration parameters θ and priors π(θ).

θ	π(θ)	Description
$\theta_{1}$	U(60,190)	Initial number of exposed (infected but not infectious) agents at the beginning of the simulation
$\theta_{2}$	U(0.03,0.1)	Base hourly probability of transmission between one infectious and one susceptible person occupying the same location
$\theta_{3}$	U(0,0.3)	Magnitude of seasonality effect
$\theta_{4}$	U(0.5,1)	Per person probability of infection scaling factor due to ratio of infectious versus susceptible people in a location
$\theta_{5}$	U(0.2,0.7)	Effective infectivity during isolation in household
$\theta_{6}$	U(0.1,0.7)	Effective infectivity during isolation in nursing home
$\theta_{7}$	U(300,700)	Simulation time (hrs) corresponding to March 27, 2020
$\theta_{8}$	U(0.1,0.8)	Reduction in out of household activities
$\theta_{9}$	U(0.01,0.3)	Reduction in transmission due to individual protective behaviors

## References

[R1] Alvi A, Ru B, Calliess J-P, Roberts S, & Osborne MA (2019). Asynchronous batch Bayesian optimisation with improved local penalisation. In International Conference on Machine Learning, pp. 253–262.

[R2] Ankenman B, Nelson BL, & Staum J (2010). Stochastic kriging for simulation metamodeling. Operations Research, 58 (2), 371–382.

[R3] Audet C, Bigeon J, Cartier D, Le Digabel S, & Salomon L (2021). Performance indicators in multiobjective optimization. European Journal of Operational Research, 292 (2), 397–422.

[R4] Azimi J, Fern A, & Fern XZ (2010). Batch Bayesian optimization via simulation matching. In Advances in Neural Information Processing Systems, pp. 109–117.

[R5] Baker E, Barbillon P, Fadikar A, Gramacy RB, Herbei R, Higdon D, Huang J, Johnson LR, Ma P, Mondal A, (2022). Analyzing stochastic computer models: A review with opportunities. Statistical Science, 37 (1), 64–89.

[R6] Balandat M, Karrer B, Jiang D, Daulton S, Letham B, Wilson AG, & Bakshy E (2020). BoTorch: A framework for efficient Monte-Carlo Bayesian optimization. In Advances in Neural Information Processing Systems, pp. 21524–21538.

[R7] Bendtsen C (2012). pso: Particle swarm optimization. R package version 1.0.3.

[R8] Binois M (2015). Uncertainty quantification on Pareto fronts and high-dimensional strategies in Bayesian optimization, with applications in multi-objective automotive design. Ph.D. thesis, Mines Saint-Etienne, EMSE.

[R9] Binois M, & Gramacy RB (2021). hetGP: Heteroskedastic Gaussian process modeling and sequential design in R. Journal of Statistical Software, 98 (13), 1–44.

[R10] Binois M, & Picheny V (2019). GPareto: An R package for Gaussian-process-based multi-objective optimization and analysis. Journal of Statistical Software, 89 (8), 1–30.

[R11] Binois M, Picheny V, Taillandier P, & Habbal A (2020). The Kalai-Smorodinsky solution for many-objective Bayesian optimization. Journal of Machine Learning Research, 21 (150), 1–42.34305477

[R12] Binois M, Gramacy RB, & Ludkovski M (2018). Practical heteroscedastic Gaussian process modeling for large simulation experiments. Journal of Computational and Graphical Statistics, 27 (4), 808–821.

[R13] Binois M, Huang J, Gramacy RB, & Ludkovski M (2019). Replication or exploration? Sequential design for stochastic simulation experiments. Technometrics, 61 (1), 7–23.

[R14] Bischl B, Richter J, Bossek J, Horn D, Thomas J, & Lang M (2017). mlrMBO: A modular framework for model-based optimization of expensive black-box functions..

[R15] Bischl B, Wessing S, Bauer N, Friedrichs K, & Weihs C (2014). MOI-MBO: multiobjective infill for parallel model-based optimization. In Learning and Intelligent Optimization, pp. 173–186.

[R16] Cajka JC, Cooley PC, & Wheaton WD (2010). Attribute assignment to a synthetic population in support of agent-based disease modeling. Methods report (RTI Press), 19 (1009), 1–14. 22577617 10.3768/rtipress.2010.mr.0019.1009 PMC3347710

[R17] Chevalier C, Bect J, Ginsbourger D, Vazquez E, Picheny V, & Richet Y (2014). Fast parallel kriging-based stepwise uncertainty reduction with application to the identification of an excursion set. Technometrics, 56 (4), 455–465.

[R18] Chevalier C, & Ginsbourger D (2013). Fast computation of the multi-points expected improvement with applications in batch selection. In Learning and Intelligent Optimization, pp. 59–69.

[R19] Chevalier C, Ginsbourger D, & Emery X (2014). Corrected kriging update formulae for batch-sequential data assimilation. In Mathematics of Planet Earth, pp. 119–122.

[R20] City of Chicago (2022). Data Portal..

[R21] Clark CE (1961). The greatest of a finite set of random variables. Operations Research, 9 (2), 145–162.

[R22] Cornuejols G, & Tütüncü R (2006). Optimization methods in finance, Vol. 5. Cambridge University Press.

[R23] Daulton S, Balandat M, & Bakshy E (2020). Differentiable expected hypervolume improvement for parallel multi-objective Bayesian optimization. In Advances in Neural Information Processing Systems, pp. 9851–9864.

[R24] Daulton S, Balandat M, & Bakshy E (2021). Parallel Bayesian optimization of multiple noisy objectives with expected hypervolume improvement. In Advances in Neural Information Processing Systems, pp. 2187–2200.

[R25] Daxberger EA, & Low BKH (2017). Distributed batch Gaussian process optimization. In International Conference on Machine Learning, pp. 951–960.

[R26] De Ath G, Everson RM, Rahat AA, & Fieldsend JE (2021). Greed is good: Exploration and exploitation trade-offs in Bayesian optimisation. ACM Transactions on Evolutionary Learning and Optimization, 1 (1), 1–22.

[R27] Deb K, Pratap A, Agarwal S, & Meyarivan T (2002). A fast and elitist multiobjective genetic algorithm: NSGA-II. Evolutionary Computation, IEEE Transactions on, 6 (2), 182–197.

[R28] Desautels T, Krause A, & Burdick JW (2014). Parallelizing exploration-exploitation tradeoffs in Gaussian process bandit optimization. Journal of Machine Learning Research, 15 (1), 3873–3923.

[R29] Emmerich MT, & Deutz AH (2018). A tutorial on multiobjective optimization: fundamentals and evolutionary methods. Natural Computing, 17 (3), 585–609. 30174562 10.1007/s11047-018-9685-y PMC6105305

[R30] Emmerich MT, Deutz AH, & Klinkenberg JW (2011). Hypervolume-based expected improvement: Monotonicity properties and exact computation. In Evolutionary Computation (CEC), 2011 IEEE Congress on, pp. 2147–2154.

[R31] Emmerich MT, Yang K, & Deutz AH (2020). Infill criteria for multiobjective Bayesian optimization. In High-Performance Simulation-Based Optimization, pp. 3–16. Springer.

[R32] Emmerich M, Giannakoglou K, & Naujoks B (2006). Single-and multiobjective evolutionary optimization assisted by Gaussian random field metamodels. Evolutionary Computation, IEEE Transactions on, 10 (4), 421–439.

[R33] Eriksson D, Pearce M, Gardner J, Turner RD, & Poloczek M (2019). Scalable global optimization via local Bayesian optimization. In Advances in Neural Information Processing Systems, pp. 5496–5507.

[R34] Eriksson D, & Poloczek M (2021). Scalable constrained Bayesian optimization. In Artificial Intelligence and Statistics, pp. 730–738.

[R35] Forrester A, Sobester A, & Keane A (2008). Engineering design via surrogate modelling: a practical guide. John Wiley & Sons.

[R36] Frazier PI (2018). Bayesian optimization. In Recent Advances in Optimization and Modeling of Contemporary Problems, pp. 255–278. INFORMS.

[R37] Garnett R (2022). Bayesian Optimization. Cambridge University Press.

[R38] Ginsbourger D (2018). Sequential Design of Computer Experiments, pp. 1–9. John Wiley & Sons.

[R39] Ginsbourger D, Le Riche R, & Carraro L (2010). Kriging is well-suited to parallelize optimization. In Computational Intelligence in Expensive Optimization Problems, pp. 131–162. Springer.

[R40] Goldberg PW, Williams CK, & Bishop CM (1998). Regression with input-dependent noise: A Gaussian process treatment. In Advances in Neural Information Processing Systems, pp. 493–499.

[R41] Gonzalez J, Dai Z, Hennig P, & Lawrence N (2016). Batch Bayesian optimization via local penalization. In Artificial Intelligence and Statistics, pp. 648–657.

[R42] Gonzalez SR, Jalali H, & Van Nieuwenhuyse I (2020). A multiobjective stochastic simulation optimization algorithm. European Journal of Operational Research, 284 (1), 212–226.

[R43] Goubier T, Rakowsky N, & Harig S (2020). Fast tsunami simulations for a real-time emergency response flow. In 2020 IEEE/ACM HPC for Urgent Decision Making (UrgentHPC), pp. 21–26.

[R44] Gramacy RB, & Lee HKH (2011). Optimization under unknown constraints. In Bayesian Statistics 9, pp. 229–256. Oxford University Press.

[R45] Gramacy RB (2020). Surrogates: Gaussian Process Modeling, Design, and Optimization for the Applied Sciences. CRC Press.

[R46] Gramacy RB, & Lee HK (2009). Adaptive design and analysis of supercomputer experiments. Technometrics, 51 (2), 130–145.

[R47] Groves M, & Pyzer-Knapp EO (2018). Efficient and scalable batch Bayesian optimization using K-means..

[R48] Guerreiro AP, & Fonseca CM (2016). Hypervolume Sharpe-ratio indicator: Formalization and first theoretical results. In International Conference on Parallel Problem Solving from Nature, pp. 814–823.

[R49] Guerreiro AP, & Fonseca CM (2020). An analysis of the Hypervolume Sharpe-Ratio Indicator. European Journal of Operational Research, 283 (2), 614–629.

[R50] Gupta S, Shilton A, Rana S, & Venkatesh S (2018). Exploiting strategy-space diversity for batch Bayesian optimization. In Artificial Intelligence and Statistics, pp. 538–547.

[R51] Haftka RT, Villanueva D, & Chaudhuri A (2016). Parallel surrogate-assisted global optimization with expensive functions-a survey. Structural and Multidisciplinary Optimization, 54 (1), 3–13.

[R52] Hennig P, & Schuler CJ (2012). Entropy search for information-efficient global optimization. Journal of Machine Learning Research, 13 (57), 1809–1837.

[R53] Hernández-Lobato JM, Requeima J, Pyzer-Knapp EO, & Aspuru-Guzik A (2017). Parallel and distributed Thompson sampling for large-scale accelerated exploration of chemical space. In International Conference on Machine Learning, pp. 1470–1479.

[R54] Hoffman MD, Brochu E, & de Freitas N (2011). Portfolio allocation for Bayesian optimization. In Uncertainty in Artificial Intelligence, pp. 327–336.

[R55] Hunter SR, Applegate EA, Arora V, Chong B, Cooper K, Rincón-Guevara O, & Vivas-Valencia C (2019). An introduction to multi-objective simulation optimization. ACM Transactions on Modeling and Computer Simulation (TOMACS), 29 (1), 1–36.

[R56] Hutter F, Hoos HH, & Leyton-Brown K (2012). Parallel algorithm configuration. In Learning and Intelligent Optimization, pp. 55–70.

[R57] Janusevskis J, Le Riche R, Ginsbourger D, & Girdziusas R (2012). Expected improvements for the asynchronous parallel global optimization of expensive functions: Potentials and challenges. In Learning and Intelligent Optimization, pp. 413–418.

[R58] Jones D, Schonlau M, & Welch W (1998). Efficient global optimization of expensive black-box functions. Journal of Global Optimization, 13 (4), 455–492.

[R59] Kandasamy K, Krishnamurthy A, Schneider J, & Póczos B (2018). Parallelised Bayesian optimisation via Thompson sampling. In Artificial Intelligence and Statistics, pp. 133–142.

[R60] LeCun Y, Bottou L, Bengio Y, & Haffner P (1998). Gradient-based learning applied to document recognition. Proceedings of the IEEE, 86 (11), 2278–2324.

[R61] Letham B, Karrer B, Ottoni G, & Bakshy E (2019). Constrained Bayesian optimization with noisy experiments. Bayesian Analysis, 14 (2), 495–519.

[R62] Lukovic MK, Tian Y, & Matusik W (2020). Diversity-guided multi-objective Bayesian optimization with batch evaluations. In Advances in Neural Information Processing Systems, pp. 17708–17720.

[R63] Lyu W, Yang F, Yan C, Zhou D, & Zeng X (2018). Batch Bayesian optimization via multi-objective acquisition ensemble for automated analog circuit design. In International Conference on Machine Learning, pp. 3306–3314.

[R64] Macal CM, Collier NT, Ozik J, Tatara ER, & Murphy JT (2018). ChiSIM: An agent-based simulation model of social interactions in a large urban area. In 2018 Winter Simulation Conference (WSC), pp. 810–820.

[R65] Mandel J, Vejmelka M, Kochanski A, Farguell A, Haley J, Mallia D, & Hilburn K (2019). An interactive data-driven HPC system for forecasting weather, wildland fire, and smoke. In 2019 IEEE/ACM HPC for Urgent Decision Making (UrgentHPC), pp. 35–44.

[R66] Marmin S, Chevalier C, & Ginsbourger D (2015). Differentiating the multipoint expected improvement for optimal batch design. In International Workshop on Machine Learning, Optimization and Big Data, pp. 37–48.

[R67] Mersmann O (2020). mco: Multiple Criteria Optimization Algorithms and Related Functions. R package version 1.15.6.

[R68] Mockus J, Tiesis V, & Zilinskas A (1978). The application of Bayesian methods for seeking the extremum. Towards Global Optimization, 2 (117–129), 2.

[R69] Moss HB, Leslie DS, Gonzalez J, & Rayson P (2021). GIBBON: General-purpose information-based Bayesian optimisation. Journal of Machine Learning Research, 22 (235), 1–49.

[R70] Mutny M, & Krause A (2018). Efficient high dimensional Bayesian optimization with additivity and quadrature Fourier features. In Advances in Neural Information Processing Systems, pp. 9005–9016.

[R71] Nguyen V, Rana S, Gupta SK, Li C, & Venkatesh S (2016). Budgeted batch Bayesian optimization. In 2016 IEEE 16th International Conference on Data Mining (ICDM), pp. 1107–1112.

[R72] Oh C, Gavves E, & Welling M (2018). BOCK: Bayesian optimization with cylindrical kernels. In International Conference on Machine Learning, pp. 3868–3877.

[R73] Ozik J, Wozniak JM, Collier N, Macal CM, & Binois M (2021). A population data-driven workflow for COVID-19 modeling and learning. The International Journal of High Performance Computing Applications, 35 (5), 483–499.

[R74] Paria B, Kandasamy K, & Póczos B (2020). A flexible framework for multi-objective Bayesian optimization using random scalarizations. In Uncertainty in Artificial Intelligence, pp. 766–776.

[R75] Parr JM (2012). Improvement Criteria for Constraint Handling and Multiobjective Optimization. Ph.D. thesis, University of Southampton.

[R76] Pearce MAL, Poloczek M, & Branke J (2022). Bayesian optimization allowing for common random numbers. Operations Research, 70 (6), 3457–3472.

[R77] Picheny V, Green DG, & Roustant O (2021). DiceOptim: Kriging-Based Optimization for Computer Experiments. R package version 2.1.1.

[R78] Poloni C, Giurgevich A, Onesti L, & Pediroda V (2000). Hybridization of a multiobjective genetic algorithm, a neural network and a classical optimizer for a complex design problem in fluid dynamics. Computer Methods in Applied Mechanics and Engineering, 186 (2), 403–420.

[R79] Ponweiser W, Wagner T, & Vincze M (2008). Clustered multiple generalized expected improvement: A novel infill sampling criterion for surrogate models. In Evolutionary Computation (CEC), 2008 IEEE Congress on, pp. 3515–3522.

[R80] R Core Team (2023). R: A Language and Environment for Statistical Computing. R Foundation for Statistical Computing, Vienna, Austria.

[R81] Rasmussen CE, & Williams C (2006). Gaussian Processes for Machine Learning. MIT Press.

[R82] Rojas-Gonzalez S, & Van Nieuwenhuyse I (2020). A survey on kriging-based infill algorithms for multiobjective simulation optimization. Computers & Operations Research, 116, 104869.

[R83] Rontsis N, Osborne MA, & Goulart PJ (2020). Distributionally robust optimization techniques in batch Bayesian optimization. Journal of Machine Learning Research, 21 (149), 1–26.34305477

[R84] Roustant O, Ginsbourger D, & Deville Y (2012). DiceKriging, DiceOptim: Two R packages for the analysis of computer experiments by kriging-based metamodeling and optimization. Journal of Statistical Software, 51 (1), 1–55.23504300

[R85] Rutter CM, Ozik J, DeYoreo M, & Collier N (2019). Microsimulation model calibration using incremental mixture approximate Bayesian computation. The Annals of Applied Statistics, 13 (4), 2189–2212. 34691351 10.1214/19-aoas1279 PMC8534811

[R86] Schonlau M, Welch WJ, & Jones DR (1998). Global versus local search in constrained optimization of computer models. Lecture Notes-Monograph Series, 34, 11–25.

[R87] Shahriari B, Swersky K, Wang Z, Adams RP, & de Freitas N (2016). Taking the human out of the loop: A review of Bayesian optimization. Proceedings of the IEEE, 104 (1), 148–175.

[R88] Sóbester A, Leary SJ, & Keane AJ (2004). A parallel updating scheme for approximating and optimizing high fidelity computer simulations. Structural and Multidisciplinary Optimization, 27 (5), 371–383.

[R89] Srinivas N, Krause A, Kakade S, & Seeger M (2010). Gaussian process optimization in the bandit setting: no regret and experimental design. In International Conference on Machine Learning, pp. 1015–1022.

[R90] Svenson JD (2011). Computer Experiments: Multiobjective Optimization and Sensitivity Analysis. Ph.D. thesis, The Ohio State University.

[R91] Tran A, Sun J, Furlan JM, Pagalthivarthi KV, Visintainer RJ, & Wang Y (2019). pBO-2GP-3B: A batch parallel known/unknown constrained Bayesian optimization with feasibility classification and its applications in computational fluid dynamics. Computer Methods in Applied Mechanics and Engineering, 347, 827–852.

[R92] Villemonteix J, Vazquez E, Sidorkiewicz M, & Walter E (2009a). Global optimization of expensive-to-evaluate functions: an empirical comparison of two sampling criteria. Journal of Global Optimization, 43 (2), 373–389.

[R93] Villemonteix J, Vazquez E, & Walter E (2009b). An informational approach to the global optimization of expensive-to-evaluate functions. Journal of Global Optimization, 44 (4), 509–534.

[R94] Wang H, van Stein B, Emmerich M, & Back T (2017). A new acquisition function for Bayesian optimization based on the moment-generating function. In 2017 IEEE International Conference on Systems, Man, and Cybernetics (SMC), pp. 507–512.

[R95] Wang J, Clark SC, Liu E, & Frazier PI (2020). Parallel Bayesian global optimization of expensive functions. Operations Research, 68 (6), 1850–1865.

[R96] Wang K, Pleiss G, Gardner J, Tyree S, Weinberger KQ, & Wilson AG (2019). Exact Gaussian processes on a million data points. In Advances in Neural Information Processing Systems, pp. 14648–14659.

[R97] Wang Z, Gehring C, Kohli P, & Jegelka S (2018). Batched large-scale Bayesian optimization in high-dimensional spaces. In Artificial Intelligence and Statistics, pp. 745–754.

[R98] Wang Z, & Jegelka S (2017). Max-value entropy search for efficient Bayesian optimization. In International Conference on Machine Learning, pp. 3627–3635.

[R99] Wilson J, Hutter F, & Deisenroth M (2018). Maximizing acquisition functions for Bayesian optimization. In Advances in Neural Information Processing Systems, pp. 9884–9895.

[R100] Wu J, & Frazier P (2016). The parallel knowledge gradient method for batch Bayesian optimization. In Advances in Neural Information Processing Systems, pp. 3134–3142.

[R101] Wu J, Poloczek M, Wilson AG, & Frazier P (2017). Bayesian optimization with gradients. In Advances in Neural Information Processing Systems, pp. 5273–5284.

[R102] Yang K, Emmerich M, Deutz A, & Fonseca CM (2017). Computing 3-D expected hypervolume improvement and related integrals in asymptotically optimal time. In International Conference on Evolutionary Multi-Criterion Optimization, pp. 685–700.

[R103] Yevseyeva I, Guerreiro AP, Emmerich MT, & Fonseca CM (2014). A portfolio optimization approach to selection in multiobjective evolutionary algorithms. In International Conference on Parallel Problem Solving from Nature, pp. 672–681.

[R104] Zhang B, Gramacy RB, Johnson L, Rose KA, & Smith E (2022). Batch-sequential design and heteroskedastic surrogate modeling for delta smelt conservation. The Annals of Applied Statistics, 16 (2), 816–842.

[R105] Zhang J, Ma Y, Yang T, & Liu L (2017). Estimation of the Pareto front in stochastic simulation through stochastic kriging. Simulation Modelling Practice and Theory, 79, 69–86.

[R106] Zhang Q, Liu W, Tsang E, & Virginas B (2010). Expensive multiobjective optimization by MOEA/D with Gaussian process model. Evolutionary Computation, IEEE Transactions on, 14 (3), 456–474.

